# A Gold-PROTAC Degrades the Oncogenic Tyrosine Kinase MERTK: Insights into the Degradome from a Steady-State System

**DOI:** 10.1021/acschembio.5c00860

**Published:** 2026-01-05

**Authors:** Sophie R. Thomas, Thomas Iellici, Mihyun Park, Elisabeth Klaus, Andrea Bileck, Christopher Gerner, Samuel M. Meier-Menches, Angela Casini

**Affiliations:** † Chair of Medicinal and Bioinorganic Chemistry, Department of Chemistry, School of Natural Sciences, 9184 Technical University of Munich, Lichtenbergstr. 4, 85748 Garching, Germany; ‡ Institute of Inorganic Chemistry, University of Vienna, Waehringer Str. 42, 1090 Vienna, Austria; § Institute of Analytical Chemistry, University of Vienna, Waehringer Str. 38, 1090 Vienna, Austria; ∥ Doctoral School in Chemistry, University of Vienna, Waehringer Str. 38, 1090 Vienna, Austria; ⊥ Joint Metabolome Facility, Medical University of Vienna and University of Vienna, Waehringer Str. 38, 1090 Vienna, Austria

## Abstract

Proteolysis targeting chimeras (PROTACs) are bifunctional molecules designed to induce the degradation of specific proteins within a cell. While most PROTACs are noncovalent interactors, covalent PROTACs may benefit from improved selectivity and pharmacodynamics, yet remain largely understudied. Here, a covalent gold-based PROTAC (**AuPROTAC**) was synthesized, featuring a Au­(III)-warhead, known to induce cysteine-arylation in a gold-templated two-step mechanism, linked to a cereblon binding moiety. The degradome of the **AuPROTAC** was characterized by establishing a cycloheximide chase assay in a nonproliferative steady-state HL-60 cell culture, enabling the identification of PROTAC degradation targets uncoupled from confounding effects originating from cell-cycle-dependent translational patterns. The method was verified using the known SMARCA2 and PBRM1-degrader ACBI2. **AuPROTAC** could degrade the oncogenic tyrosine kinase MERTK and the thioredoxin-like 1 protein TXNL1. Their degradation was successfully rescued by proteasome inhibition. Proteome-wide degradation selectivity was further characterized by ranking the degraded targets according to the reduction extent of their protein half-lives. Interestingly, the **AuPROTAC** degraded a relatively limited number of proteins (95) when compared to ACBI2 (221).

## Graphical abstract

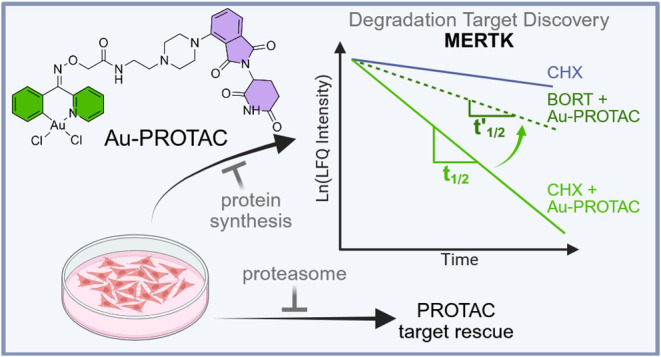

## Introduction

Targeted protein degradation is an innovative strategy in drug discovery and chemical biology, facilitating the polyubiquitination and subsequent degradation of a target protein by exploiting the induced proximity of a Ubiquitin E3 ligase to the target.^1–3^ Small molecules that can induce targeted protein degradation are classified as proteolysis targeting chimeras (PROTACs) or molecular glues.^4^ PROTACs are heterobifunctional compounds, acting via a catalytic, substoichiometric mechanism to eliminate disease-causing proteins from the cell. The PROTAC design entails tethering a ligand, often a pharmacological inhibitor, that binds a selected (onco)­protein, to a ligand that recruits the E3 ubiquitin ligase via a linker (Figure 1A). The judicious choice of the latter is essential to form a stable and functional ternary complex. Molecular glues enhance the interaction between a target protein and the E3 ligase in a single functional compound, exemplified by thalidomide and its analogues.^5^ PROTACs deplete the target proteins by hijacking the cellular protein destruction machinery, mainly the ubiquitin-proteasome system (UPS), but lysosome^6^- and autophagy^7^-targeting are also known strategies. Consequently, the mechanism of action distinguishes PROTACs from conventional enzyme inhibitors, as the latter only block a protein’s activity without degrading it. Targeted protein degradation offers potential advantages, including reduced dosing requirements and prolonged effects. Additionally, PROTACs have shown great promise due to their ability to target “undruggable” proteins, including transcription factors or protein–protein interaction mediators.^8^ PROTACs have been shown to improve anticancer immunotherapy by degrading specific proteins,^9^ and the first designed molecular glue was recently approved in a combination treatment.^10^

**Figure 1:**
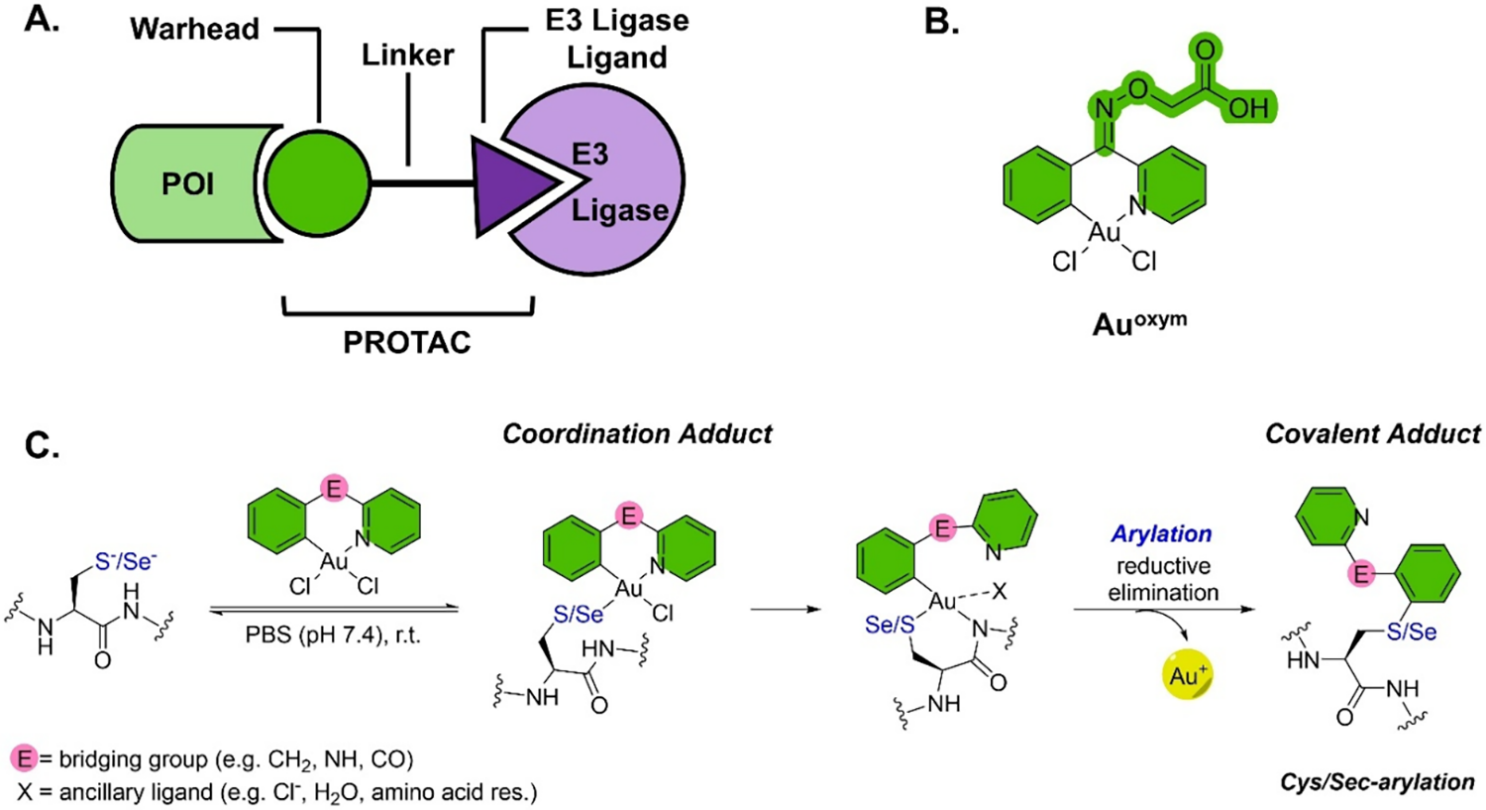
(A) Schematic of the PROTAC design; (B) chemical structure of cyclometalated Au­(III) C^N compound **Au** ^**oxym**^ used as warhead in this study; (C) two-step mechanism of the Au­(III)-templated cross-coupling reaction. The arylation of cysteine (and seleno-cysteine) residues is accomplished by reductive elimination.

Currently, the majority of PROTACs use reversible noncovalent ligands for both the target protein of interest (POI) and the E3 ligase. This implies that PROTACs can act catalytically,^11^ which reduces systemic drug exposure and potential adverse effects. Nevertheless, recently, there have been successful examples of PROTACs using covalent warheads that, while acting stoichiometrically, have degraded a range of targets.^12–15^ For example, ERK1/2,^16^ Bruton tyrosine kinase (BTK),^17,18^ and KRASG12C^19^ have been reported to be degraded by covalent PROTACs. Typically, protein degradation is driven by the reversible binding to the target, prior to covalent bond formation,^20^ and the subsequent formation of a ternary complex with the E3 ligase.

The covalent binding property of the PROTAC to the target protein could translate into more favorable pharmacokinetic properties, such as enhanced cell permeability and reduced susceptibility to efflux, ultimately leading to greater target degradation efficacy.^13,21,22^ Thanks to the ability of covalent warheads to target nucleophilic amino acids even in shallow binding sites previously considered ‘hard-to-drug’, covalent PROTACs could enable the targeting of less tractable allosteric binding sites. The discovery of pharmacologically useful covalent binding sites of electrophilic compounds is facilitated by chemoproteomic platforms.^23^ In an electrophile-first approach, these technologies enable the proteome-wide identification of binding sites and selectivity profiling of covalent ligands. In fact, it has been reported that the strongest binding interactions per atom with a certain target are associated with metals, anions, and small ligands (10–20 atoms) that form covalent bonds.^22^ Finally, sustained target engagement in covalent binders can result in less frequent dosing, and even the potential to evade resistance mutations that affect reversible inhibitors,^24,25^ thereby preventing disease relapse. Despite these advantages, the loss of catalytic activity in irreversible covalent degraders has prompted the development of *reversible* covalent PROTACs,^13,20,26^ which combine the benefits of covalent bond formation with substoichiometric target turnover.

Metal-based anticancer agents are a versatile class of pharmacologically active compounds and include several metal families, *e.g.*, based on platinum, ruthenium, or gold, among others.^27,28^ A considerable number of metallodrugs can be categorized as (reversible) covalent inhibitors, because their mode of action relies on direct coordination of the metal to the target.^29–31^ In contrast to the well-known binding via metal coordination, we recently reported on a cyclometalated Au­(III) complex (**Au** ^**oxym**^, Figure 1B), featuring a bidentate C^N ligand,^32^ that covalently targets the CysSec-dyad of thioredoxin reductase 1 (TXNRD1) via gold-templated bioorthogonal reactivity. This reactivity involves an unconventional two-step mechanism: first, the reversible coordination of the cyclometalated Au­(III) compound to thiolate/selenolate groups occurs, followed by the irreversible C–S/Se cross-coupling reaction of the ligand to these nucleophiles via reductive elimination (Figure 1C).^33–36^ Using combined chemoproteomic and complementary methods, we have shown that this bioorthogonal reaction allows remarkably selective targeting of TXNRD1 by the gold compound in human SW480 colon carcinoma cancer cells.^32^ TXNRD1 is part of the thioredoxin (TXN) system, one of the major cellular antioxidant pathways that control redox homeostasis.^37^ It also regulates cell growth, apoptosis, gene expression, and antioxidant defense in nearly all living cells. Targeting the activity of the thioredoxin system is considered a promising strategy in cancer treatment.^38,39^ The resulting arylation of the TXNRD1 catalytic CysSec-dyad by the gold compound leads to potent and irreversible enzyme inhibition in human colon carcinoma cancer cells.^32^

Based on these promising results, we hereby report the synthesis of the first gold-based PROTAC featuring the aforementioned cyclometalated Au­(III) C^N moiety (**Au** ^**oxym**^) as a covalent protein binder, which acts via a gold-templated aryl-transfer reaction onto thiolate groups of cysteine residues. Moreover, the analysis of the compound’s proteome-wide target degradation (degradome) has been performed. The design concept is presented in Scheme 1 and entails tethering the Au­(III) C^N warhead to the classical E3 ligase cereblon (CRBN) interacting moiety via the linker of Vepdegestrant (ARV-471).^40^ Metallo-PROTAC strategies were already reported for platinum derivatives,^41–43^ a gallium complex,^44^ and in one case, ferrocene was employed as an interesting redox-active linker.^45^ Here, the reactivity of the **AuPROTAC** compound with a model cysteine compound was first assessed by ^1^H NMR spectroscopy, as previously performed for **Au** ^**oxym**^ to evaluate its ability to template the cysteine arylation reaction.^32^ The degradome of the **AuPROTAC** was further studied in the differentiated human myeloid leukemia HL-60 cell line, which was found to robustly express CRBN, VHL, and the respective E3-Ligase machineries. A comprehensive method for degradome analysis was then established based on protein turnover analysis and benchmarked using ACBI2 (Scheme S1), an optimized degrader of the probable global transcription activator SNF2L2 (SMARCA2) and protein polybromo-1 (PBRM1).^46^ Although **AuPROTAC** did not degrade TXNRD1, as would have been expected from the **Au** ^**oxym**^ reactivity, we discovered the kinase MERTK and thioredoxin 1-like protein (TXNL1) to be degraded. Moreover, the **AuPROTAC**-induced degradome could be further assessed for differences in protein half-lives, which revealed additional targets. In contrast to a recent report,^47^ degradome analysis, as reported here, can reveal the selectivity of degradation efficiency based on the magnitude of protein half-life reduction induced by the PROTACs in the nonproliferative steady-state system. The latter enables the discovery of protein target degradation and provides a comprehensive characterization of the degradome of CRBN- and VHL-targeted PROTACs.

**Scheme 1:**
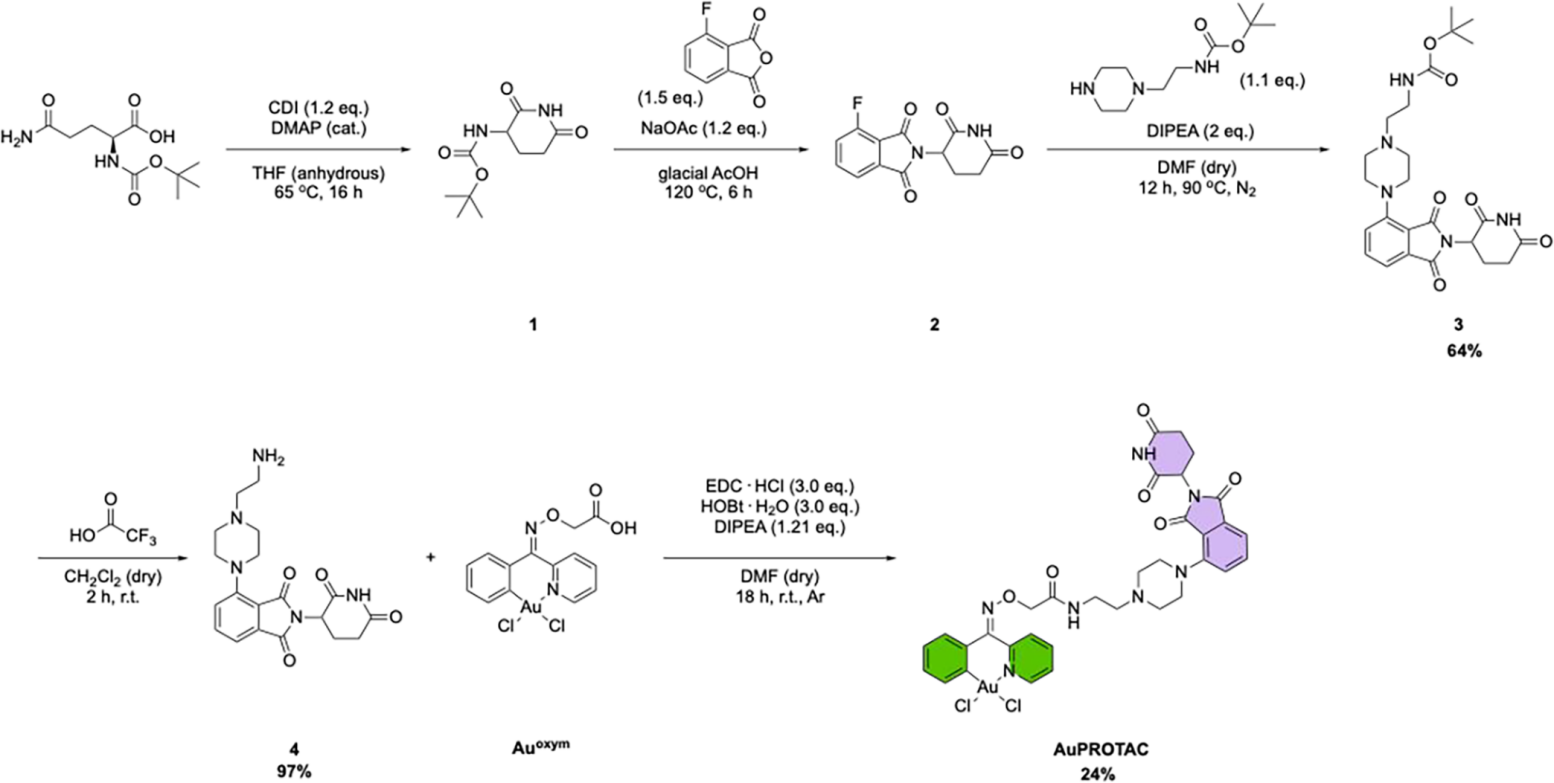
Synthesis of the Au­(III)-Based **AuPROTAC**.

## Results and Discussion

### Synthesis and Characterization

AuPROTAC was designed based on the E3 ligase ligand CRBN, using a linker similar to that of Vepdegestrant (ARV-471), with adaptations made for synthetic feasibility and length. Vepdegestrant (Scheme S1) is an estrogen receptor degrader for breast cancer, which has very recently been submitted for a New Drug Application (NDA) to the U.S. Food and Drug Administration (FDA) by Arvinas and Pfizer.^48,49^ The E3 ligase ligand was synthesized starting from **1** and **2**, based on published procedures.^50^ In detail, *N*-α-(*tert*-butoxycarbonyl)-l-glutamine (Boc-Gln) underwent cyclization in the presence of 1,1-carbonyldiimidazole (CDI) and catalytic amounts of 4-(dimethylamino)­pyridine (DMAP), forming boc-2-aminoglutarimide (**1**). A condensation reaction was then performed between **1** and 3-fluorophthalic anhydride in glacial acetic acid with sodium acetate (NaOAc) to yield **2**.^50^ Next, to connect the E3 ligase ligand to the proposed linker, **2** was reacted with *tert*-butyl (2-(piperazin-1-yl)­ethyl)­carbamate to form **3**. Thus, after deprotection of the Boc group, **4** could undergo an amide coupling reaction with **Au** ^**oxym**^ to yield the desired **AuPROTAC**. The precursors (**3** and **4**) and **AuPROTAC** were fully characterized by standard analytical methods, including ^1^H, ^13^C, and 2D NMR, high-resolution mass spectrometry (HR-MS), including (desorption) electrospray ionization (D-ESI), and elemental analysis (EA) (see Figures S1–S15).

### AuPROTAC Stability and Reactivity

The stability of **AuPROTAC** was then monitored by ^1^H NMR in DMSO-*d*_6_:D_2_O (9:1) with a spectrum measured at different time intervals over 24 h. Ligand exchange reactions with the ancillary chloride ligands can be easily monitored by a shift in the proton adjacent to the pyridyl N (α-H). As shown in Figure S16, no changes in the NMR spectra were observed over 24 h. The ability of the gold complex to arylate thiols was next tested to ensure the metal-templated reactivity remained after the addition of the PROTAC ligand to **Au** ^**oxym**^. In detail, ^1^H NMR spectra were recorded over 24 h following the addition of 3 equiv. *N*-acetyl cysteine (NAC), a model cysteine residue. As shown in Figure 2, a characteristic shift of the α-H of **AuPROTAC**, from 9.28 to 8.58 ppm (Δ = 0.7 ppm), was observed already at time 0 after addition of the amino acid to the compound, corresponding to an immediate cysteine arylation.^32,51^ Based on previous reports on other C–S cross-coupling reactions templated by different organogold-based warheads,^52^ we estimate that the reaction occurs within the very first minutes, or even seconds, depending on the experimental conditions used (*e.g*., ionic strength, pH, presence of competing nucleophiles), in a time frame not compatible with the time scale of the NMR analysis.

**Figure 2:**
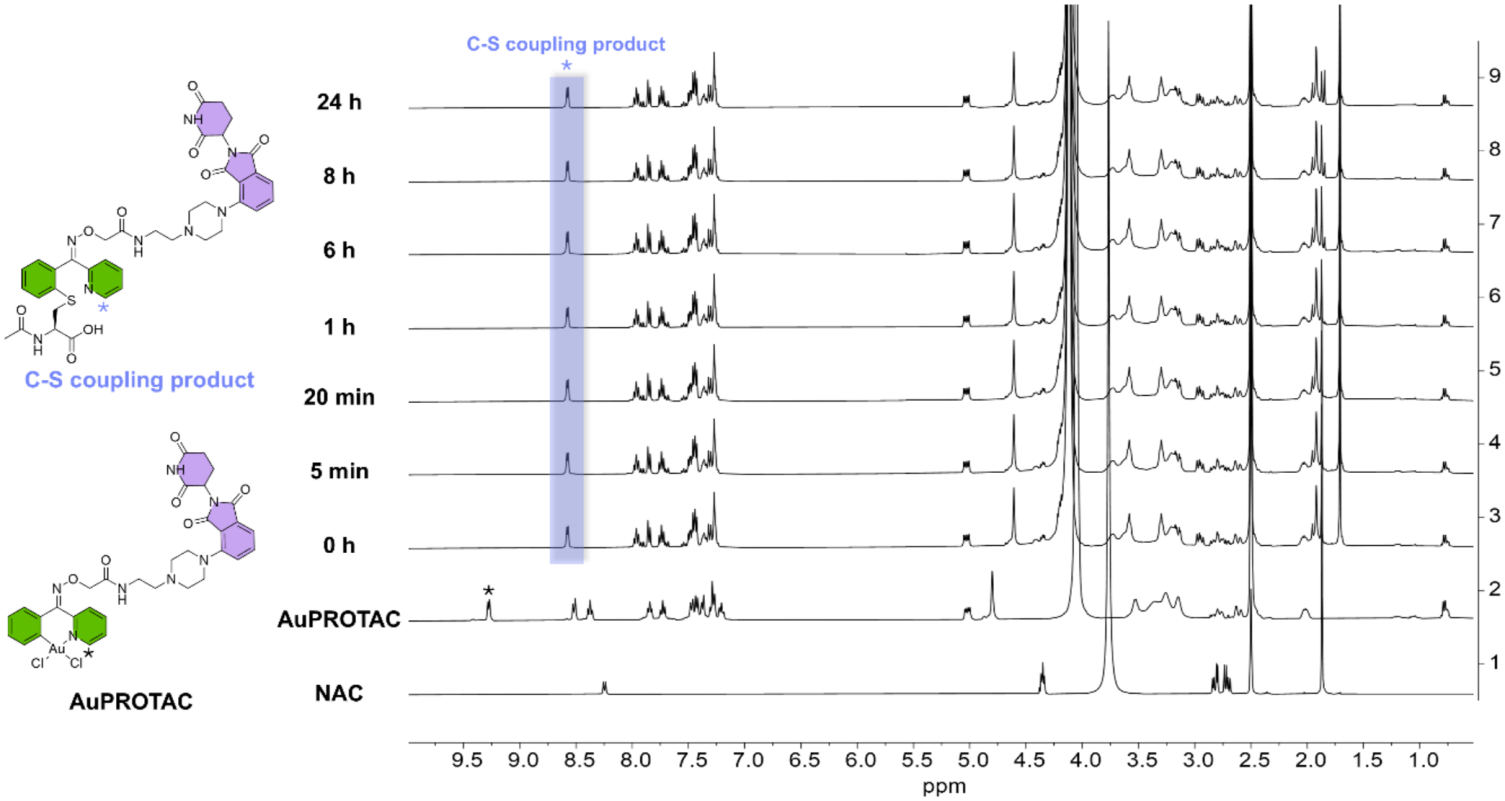
^1^H NMR spectra of **AuPROTAC** reacting with 3 equiv of NAC in DMSO-*d*_6_:D_2_O (9:1). The spectra of **AuPROTAC** and NAC alone are included as reference in the same solvent system. The characteristic shift of the α-H (*) was observed (Δ = 0.7 ppm), indicating a rapid C–S cross-coupling between **AuPROTAC** with NAC.

### Designing a Cycloheximide Chase Assay for Degradome Analysis

Proteomic analysis of PROTAC activity is typically performed by comparing the PROTAC to an inactive analogue.^53^ Here, we hypothesized that PROTAC activity can be estimated from protein turnover. Indeed, targeted protein degradation is tightly linked to protein turnover, which is a function of protein synthesis and degradation. Since conventional end point proteome profiling experiments cannot distinguish between protein down-regulation and protein degradation,^47^ dedicated methodologies were established to specifically assess protein turnover, including, among others, translation inhibition^47,54^ or pulse-chase approaches using stable isotope labeled metabolites.^55,56^ Furthermore, protein turnover can change according to dynamic cell states. With respect to PROTAC-induced proteome-wide degradation (degradome) analysis, it is important to ensure steady-state conditions in which the net change of protein levels is zero, *i.e.*, the rates of protein synthesis and degradation are equal.^55^ Such a steady-state system is largely independent of anabolism, proliferation, or cell-cycle dependent effects that are known to affect protein turnover and, therefore, protein degradation dynamics, but also avoids dilution of protein pools due to cell division.^57^ In practical terms, this requires a nonproliferative cell model system, despite proliferating cell models being the norm.^56^ In such a steady-state, blocking protein synthesis by means of a translation inhibitor, *e.g*., cycloheximide (CHX), effectively decouples protein degradation from drug-induced transcriptional or translational down-regulation. Protein degradation can then be approximated by first-order decay kinetics.^58^ Consequently, the combination of a steady-state system with a translation inhibitor seems appropriate for analyzing PROTAC-induced degradomes (cf. assumptions in Experimental Section). Target degradation can be explored by differential analysis of the time-dependent PROTAC activity in the CHX-pretreated cells. Determining protein half-lives under these conditions further enables the selective characterization of PROTAC-degraded proteins. This has the potential advantage that degraded proteins can be ranked according to the magnitude of the decrease in protein half-lives, serving as a measure of PROTAC-induced degradation efficiency. Our approach, therefore, complements a recently published method for degradome analysis^47^ and can be implemented in a PROTAC validation workflow.^53^

### Differentiated HL-60 Cells Represent a Nonproliferative Steady-State System

Following the above-mentioned consideration, we set out to identify a suitable cellular steady-state system that would express the required components of the protein degradation machinery required for the studied PROTACs. ACBI2 was used as a known PROTAC to validate the approach. While ACBI2 features a von Hippel-Lindau disease tumor suppressor (VHL) binding moiety, **AuPROTAC** contains a protein cereblon (CRBN) binding moiety. From a panel of cell lines, the differentiated acute myeloid leukemia cell line HL-60 (FAB M2) was identified as a suitable candidate system. HL-60 cells were differentiated with phorbol 12-myristate 13-acetate (PMA) over 72 h, after which they showed a clear nonproliferative steady-state phenotype, even over prolonged incubation times (Figure 3A). We have previously shown that AML cell lines can be robustly differentiated and used for proteomic perturbation studies.^59^ Moreover, differentiated HL-60 cells exhibit a characteristic adherent phenotype that can be readily confirmed by light microscopy (Figure 3B).

**Figure 3:**
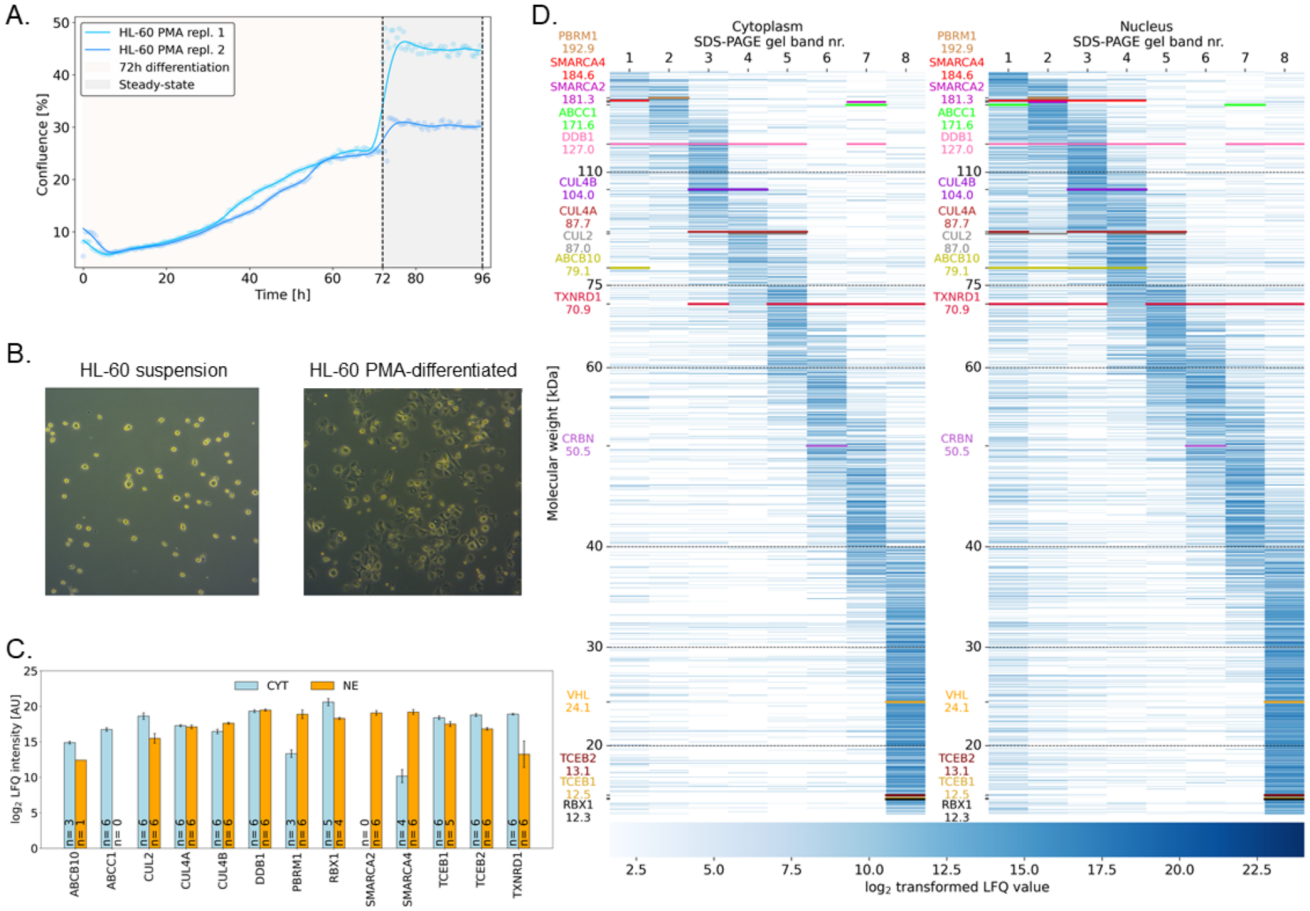
Characterization of differentiated HL-60 cells as a nonproliferative steady-state model system for degradome analysis. (A) Live-cell monitoring of confluence in 6-well plates of PMA-differentiated HL-60 cells for 72 h, followed by medium exchange and a steady-state period. (B) Light microscopy images (10× magnification) of nondifferentiated (*left*) and PMA-differentiated (*right*) HL-60 cells, highlighting the adherent phenotype upon differentiation. (C) Log2-transformed label-free quantification (LFQ) intensities of proteins of interest in the cytoplasmic (CYT) and nuclear (NE) extracts. (D) Deep proteome profiling of nondifferentiated HL-60 cells by offline prefractionation using 1D gel electrophoresis. LFQ protein intensities are shown in a heat map and the proteins of interest relevant to the PROTAC activity are highlighted.

A deep proteome analysis based on fractionation by gel electrophoresis and in-gel digestion of the proteins identified a total of 6488 proteins in 16 fractions. A data-dependent analysis strategy was followed based on label-free quantification (LFQ) proteomics. We used a nanoflow liquid chromatography-tandem mass spectrometry (nLC-MS/MS) system based on a TimsTOF Pro mass spectrometer in parallel accumulation-serial fragmentation mode^60^ throughout this study. A 90 min nLC-gradient distinctly improved the number of unique peptides compared to shorter gradients. A minimum of 1 unique peptide further increased the number of detected proteins compared to a 2-peptide search and reduced the median data variation in the data set (Figure S17A–D). The E3 ligase recruiters VHL and CRBN were successfully detected, as were components of their E3 ubiquitin ligase complexes based on cullin-RING ligases^61,62^ (Figure 3C–D). The E3 ligase complex formed with the VHL recruiter contains elongin B (TCEB2), elongin C (TCEB1), cullin-2 (CUL2), and E3 ubiquitin-protein ligase RBX1 (RBX1).^61^ The E3 ligase complex formed with the CRBN recruiter includes DNA damage-binding protein 1 (DDB1), cullin-4A (CUL4A) or cullin-4B (CUL4B), and RBX1.^62,63^ The degradation targets of ACBI2 were also identified, including SMARCA2, PBRM1, and the transcription activator BRG1 (SMARCA4).^46^ TXNRD1, the suggested degradation target of the **AuPROTAC** ^32^ was also detected. Therefore, the HL-60 cell line represents a suitable model system to discover proteome-wide protein degradation by VHL- and CRBN-type degraders.

### The Cycloheximide Chase Assay Can Be Implemented in the HL-60 Steady-State System for Degradome Analysis

Cell viability assays were then performed on the PMA-differentiated HL-60 cells to select suitable treatment concentrations (Figure S17E). First, the CHX treatment was optimized with respect to an effective translation inhibition by testing a range of concentrations (40 nM to 10 μM) over 24 h. Since translation inhibition globally reduces protein synthesis, LFQ proteome profiles should reveal a major decrease in protein intensity of a broad range of proteins. A CHX concentration of 10 μM was required to observe noticeable translation inhibition at the proteome level, *i.e*., 1980 proteins showed reduced intensity (Figure S17F). The CHX chase assay was then performed in steady-state HL-60 cells up to 8 h. To assess time-dependent protein degradation, cells were collected at the start of the chase (0 h), as well as after 0.5, 1, 2, 4, and 8 h (Figure 4A). At each time point, whole cell lysates were collected in triplicates and separately processed by LFQ proteomics. Four different conditions were carried out, including a vehicle-treated control (CON), CHX-treated cells, and CHX+PROTAC cotreated cells. A total of 5834 proteins were identified in the data set. As expected from the moderate cytotoxic effects of the CHX treatment up to 8 h (Figure S17G), apoptosis markers and caspase abundances remained constant over the incubation period (Figure S18A), indicating the absence of confounding effects of cell death. CHX+PROTAC cotreatment slightly reduced the confluence over the 8 h treatment (Figure S18B), which was addressed by normalizing the protein amount for proteolytic digestion. The presence of the proteins of interest related to the PROTAC mechanism was again confirmed (Figure S18C). The (putative) degradation targets of ACBI2 (SMARCA2 and PBRM1) and **AuPROTAC** (TXNRD1) were also observed and were not affected by the CHX treatment (Figure 4B).

**Figure 4:**
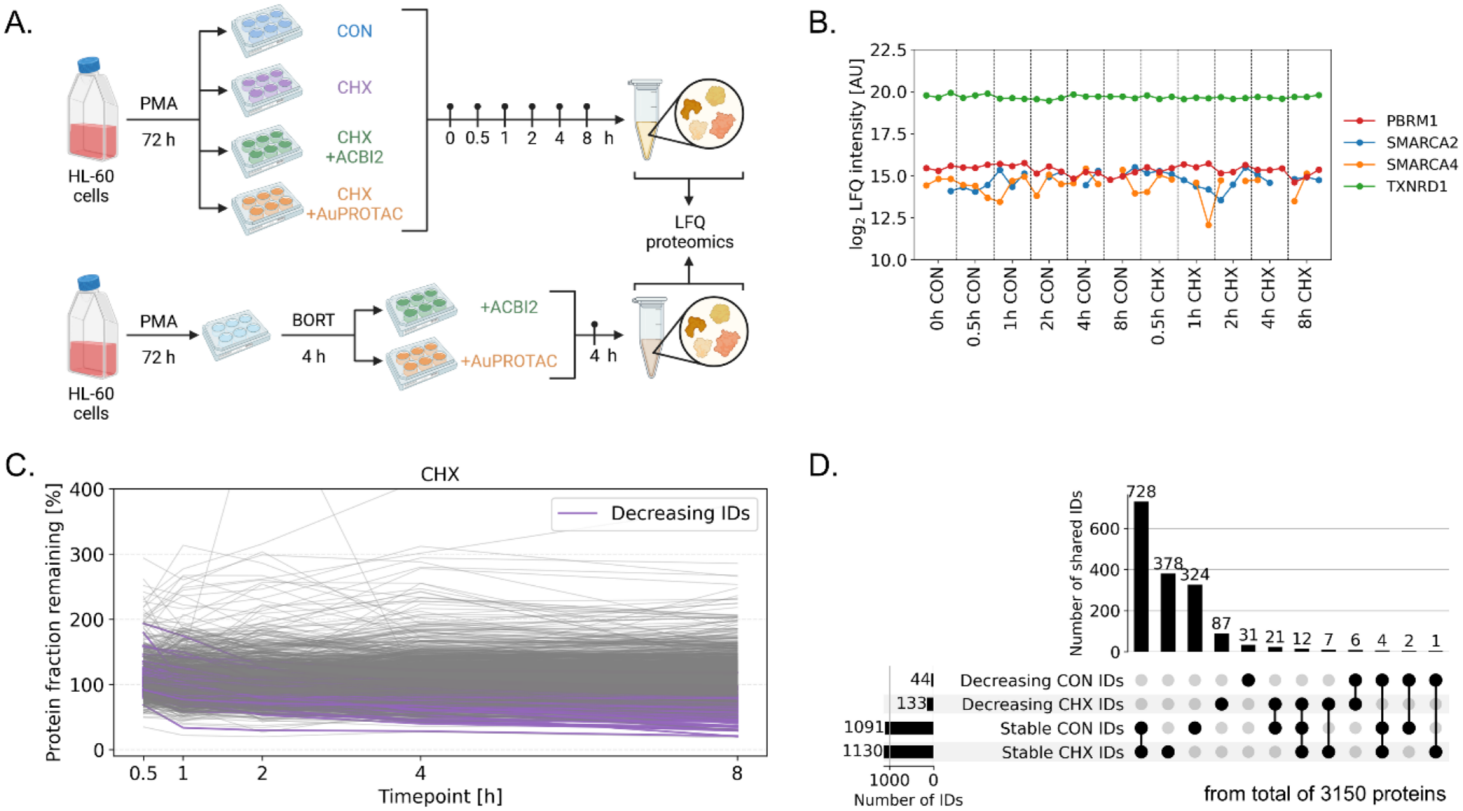
Establishing a cycloheximide (CHX) chase assay in the steady-state HL-60 cell model. (A) Experimental scheme to assess degradomes using the CHX chase assay and rescue by proteasome inhibition using bortezomib (BORT). (B) Log2-transformed protein LFQ intensities highlight the stability of putative target proteins of ACBI2 and **AuPROTAC** in CON or CHX-treated cells up to 8 h. (C) Profile plot of fully detected proteins (*N* = 3150) in CHX-treated HL-60 cells as protein fraction remaining over 8 h chase. The proteins highlighted in color show natural decay profiles. (D) Upset plot of stable and naturally decaying proteins in CON and CHX conditions.

A total of 3150 proteins (of 5834, 54%) were detected in all samples (see Experimental Section). In this set, stable proteins in the CON and CHX conditions made up 1091 and 1130 proteins (both 35%), respectively (Figure S18D). Linear LFQ-intensity values were then transformed to protein fractions remaining (PFR)^56,64^ and summarized in profile plots enabling a data set-level overview. The profile plots highlight the stability of the proteome in the CON and CHX-treated steady-state system over the investigated period of 8 h, featuring only 44 and 133 proteins, respectively, that showed decay characteristics (Figures 4C and S19A and Supporting Data 1). The minor protein changes in CON- and CHX-treated cells support a steady-state under the experimental conditions. There was little overlap in those proteins according to identity (*n* = 6, Figure 4D).

### Discovery of Targeted Protein Degradation

Degradation of target proteins can be detected in this assay through differential analysis of CHX- and CHX+PROTAC-treated proteomes after a specified chase period, followed by further verification through PROTAC-induced acceleration of first-order decay kinetics. Translation inhibition by CHX eliminates transcriptional and translational regulation of proteins, efficiently decoupling protein degradation from regulation. In a first step, the volcano plots for ACBI2 and **AuPROTAC** were used to select the most strongly degraded potential protein targets (Figure 5A,B). Those were not necessarily significantly regulated proteins, as there were only a small number of significantly regulated proteins in the volcano plots of both PROTACs, indicating the lack of translational responses to the treatment. In a second step, it was verified whether the selected proteins exhibited degradation kinetics that could be identified as degradation targets. As expected, the optimized ACBI2 induced the rapid and quantitative degradation of SMARCA2 and PBRM1 already after 30 min, while SMARCA4 remained largely unaffected (Figure 5A). Moreover, we observed that PTHR1, SRPK1, and TRIM26 were degraded in a delayed manner after 8 h (Figure S19B), indicating off-target effects of ACBI2, likely after the complete degradation of the initial target proteins. SRPK1 and TRIM26 were quantitatively degraded, whereas PTRH1 remained detectable.

**Figure 5:**
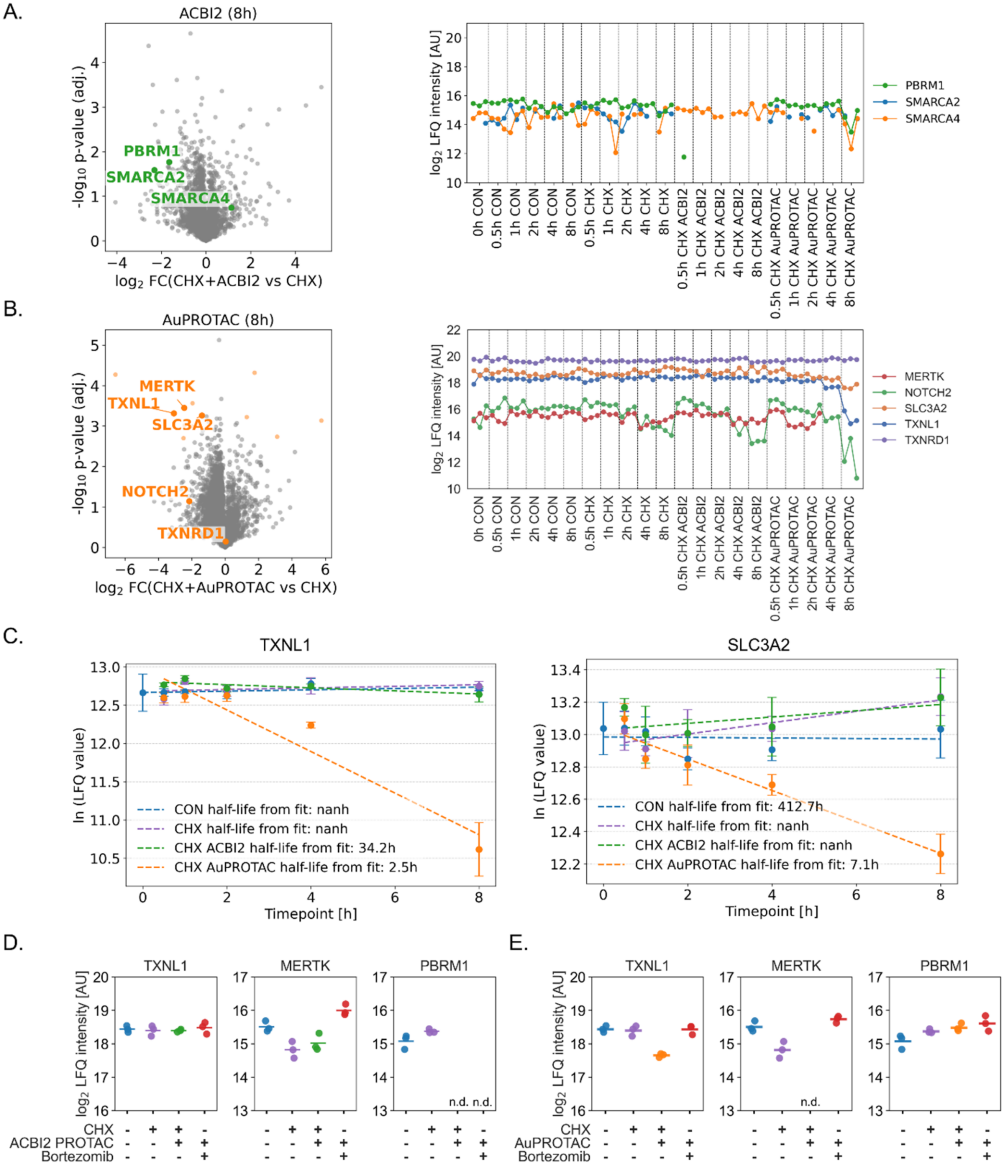
Discovery of PROTAC-induced degradation targets. Selection of the potential targets of ACBI2 (A) and **AuPROTAC** (B) by differential analysis of CHX+PROTAC vs. CHX treatment after 8 h chase in a Volcano plot and subsequent identification by degradation kinetics in profile plots. The statistical significance of protein regulation is shown in light colored dots in the Volcano plots (FDR = 0.05, S0 = 0.1). Input data for Volcano plots was imputed by left-censored random distribution. Input data for the profile plots was not imputed. (C) Estimation of protein half-life of **AuPROTAC** targets TXNL1 and SLC3A2. Verification of degradation targets using bortezomib (BORT) as a proteasome inhibitor for ACBI2 (D) and **AuPROTAC** (E) after 4 h. The untreated control (CON) cells and CHX-treated cells (4 h) served as method control.

The **AuPROTAC** quantitatively degraded the oncogenic tyrosine kinase MERTK, TXNL1, amino acid transporter heavy chain SLC3A2 (SLC3A2), and notch homologue protein 2 (NOTCH2) were partially degraded (Figure 5B–C). MERTK was shown to contribute to tumor proliferation,^65,66^ and could be degraded by conventional kinase inhibitor-based PROTACs.^67^ Of note, the kinase domain of MERTK (PDB 7M5Z) does not contain a cysteine, but is nonetheless efficiently degraded by **AuPROTAC**. Interestingly, TXNL1 was found to be strongly down-regulated^32,68^ or oxidized^69^ in other perturbation studies of different gold-based candidate drugs and; therefore, represents a likely target for **AuPROTAC**. The protein is known to exhibit redox properties and act as a redox-independent chaperone,^70^ negatively regulating apoptosis.^71^ Additionally, SLC3A2 is required for AML cell proliferation and its deletion impairs disease progression.^72^ TXNRD1 was not degraded by **AuPROTAC** in the HL-60 cells, although **Au** ^**oxym**^ was shown to target this protein in SW480 cells.^32^ This may be expected since the relatively large CRBN-recruiter and the linker moiety may affect the intracellular accumulation and subcellular distribution of the reactive warhead. Furthermore, it is known that even small structural changes in covalent metallodrugs considerably alter their target landscape.^73^ It was also previously shown in the case of kinase inhibitors that the PROTACs′ selectivity would not just be influenced by the kinase targeting moiety, but also by the entire construct.^74^

To verify the targeted degradation of these proteins, we performed an additional experiment by preincubating differentiated HL-60 cells in steady-state with bortezomib (BORT), a known and potent proteasome inhibitor, which is expected to block proteasome-mediated degradation (Figure 4A). On the one hand, degradation of PBRM1 by ACBI2 could not be rescued by proteasome inhibition due to the exceptionally fast degradation (Figure 5D). Instead, the degradation targets of **AuPROTAC** corresponding to MERTK and TXNL1 were successfully rescued by proteasome inhibition (Figure 5E), underlining that these are true degradation targets. Importantly, the degradation targets were not affected by the respective other PROTAC.

### Characterizing Degradomes by Protein Half-Life Distributions

The steady-state system remained stable for the CHX+PROTAC cotreatments as revealed in the profile plots, confirming that only a small fraction of proteins is affected by the PROTAC-induced degradation (Figures 6A and S19C). We found that CHX+ACBI2 and CHX+**AuPROTAC** treatments degraded 320 and 166 proteins, respectively (Supporting Data 1). Of those, 221 and 95 were unique to the CHX+ACBI2 and CHX+**AuPROTAC** treatments, respectively (Figure 6B and Supporting Data 2). A total of 15 proteins featured decay kinetics in all conditions, indicating CHX-dependent effects, *e.g.*, heme oxygenase 1 (HMOX1, Figure 6C). Then, protein half-lives were calculated for the naturally decaying and degraded proteins in each condition, and the distribution of the unique proteins in each condition was visualized in box plots (Figure 6D). The median of the protein half-lives for control and CHX conditions were 15.0 and 11.5 h, respectively. The ACBI2 and **AuPROTAC** conditions showed median protein half-lives of 13.3 and 10.5 h, respectively. This indicated that the PROTACs distinctly altered protein degradation dynamics.

**Figure 6:**
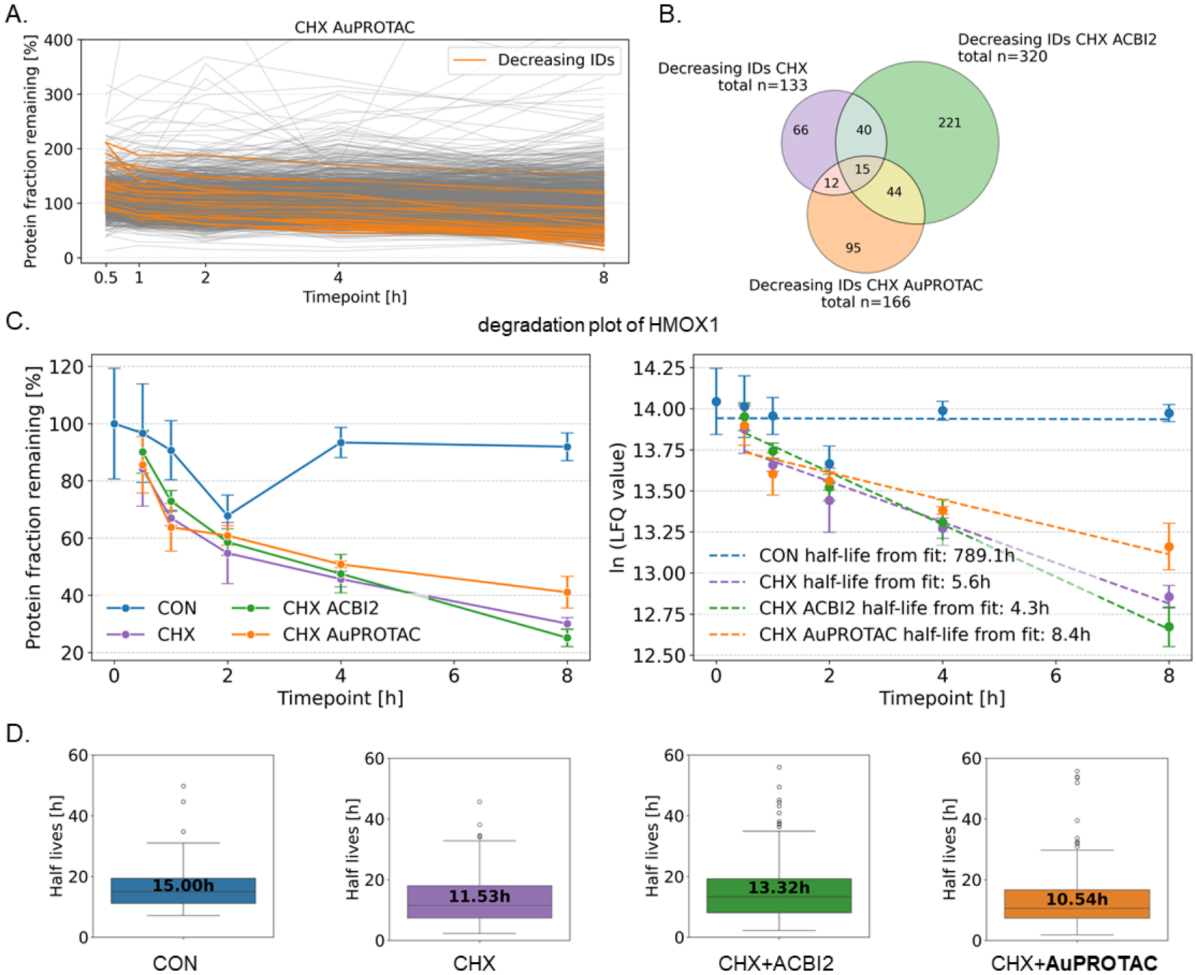
(A) Profile plot of the proteins (*N* = 3150) detected in all samples of CHX+**AuPROTAC** treated cells as protein fraction remaining. The proteins highlighted in color are continuously decreased by CHX+**AuPROTAC** across all time points (*N* = 166). (B) Venn diagram of proteins that naturally decay (CHX) or are degraded (CHX+PROTAC) in the respective treatments. (C) Heme oxygenase 1 (HMOX1) is one of the 15 proteins that show natural decay in CHX and CHX+PROTAC-treated cells. (D) Distribution of protein half-lives of proteins that naturally decay (CON (*n* = 44), CHX (*n* = 66)) and are degraded (CHX+AuPROTAC (*n* = 95) and CHX+ACBI2 (*n* = 221)). The median protein half-life [h] is shown. Estimated protein half-lives >60 h were excluded because of the maximum chase period of 8 h.

PROTAC-induced degradomes can be further quantified based on differences in protein half-lives (Δ*t*_1/2_) between the PROTAC+CHX and CHX-only conditions, as PROTACs are expected to accelerate the degradation rate of target proteins. The affected proteins can then be ranked according to the magnitude of Δ*t*_1/2_ as a measure of the potency of the PROTAC-induced degradation. This approach necessitates knowledge about protein half-lives in PROTAC+CHX and CHX conditions and is therefore, shown for the overlapping proteins between CHX and CHX+PROTAC conditions identified in the Venn diagram of Figure 6B., i.e., 27 proteins between CHX and CHX+**AuPROTAC** (Table S1) and 55 proteins between CHX and CHX+ACBI2 (Table S2). Importantly, the PROTACs decreased protein half-lives in the respective subset of shared proteins, with frequencies of 71% for ACBI2 (Figure 7A) and 78% for **AuPROTAC** (Figure 7B). The Δ*t*_1/2_ between PROTAC+CHX and CHX was further expressed in rank-based plots (Figure 7A,B). For 15 proteins, protein half-lives were available in all three conditions (see Venn diagram, Figure 6B). Among those was Filamin-A (FLNA) for which the Δ*t*_1/2_ was 22 and 11 h for **AuPROTAC** and ACBI2 treatments, respectively (Figure 7C). The Δ*t*_1/2_ of FLNA was the largest for **AuPROTAC**, while it was found in fourth position for ACBI2. Therefore, FLNA may represent a potential candidate for unspecific PROTAC degradation. Interestingly, the **AuPROTAC**, but not ACBI2, enhanced the degradation of metallothionein-1X (MT1X) with a Δ*t*_1/2_ of 6 h (Figure 7D). Metallothioneins are known to be strong binders of transition metals,^75^ including gold,^76^ and may represent an off-target effect of **AuPROTAC**. Of the shared proteins between the two PROTAC treatments, 80% showed longer half-lives in the ACBI2 treatment compared to the **AuPROTAC**. This indicates that **AuPROTAC** affects the half-lives of a smaller number of proteins, but to a stronger extent compared to the ACBI2 treatment. Overall, this approach enables characterizing the PROTAC-impact on degradomes, revealing detailed protein degradation dynamics.

**Figure 7:**
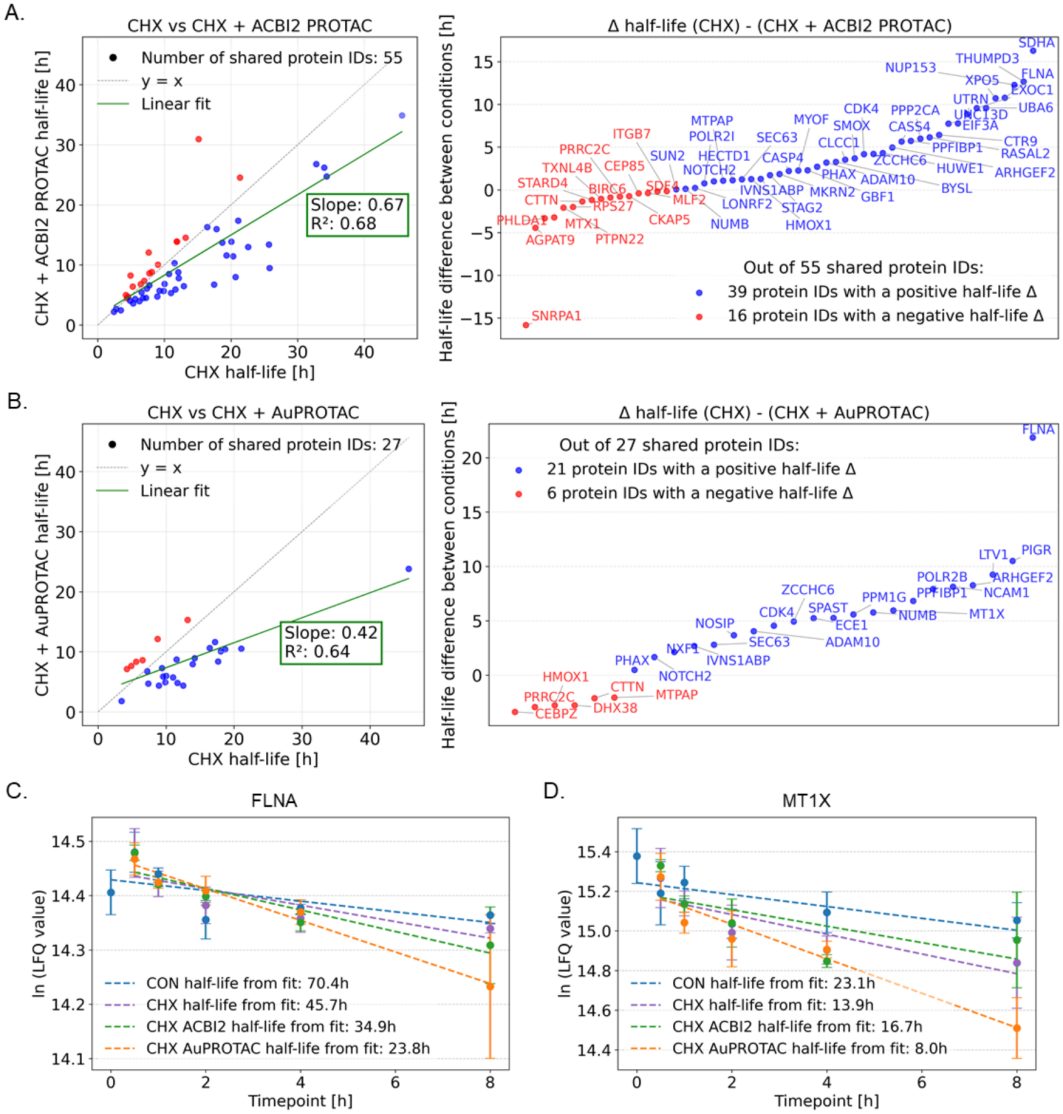
Assessment of PROTAC-induced protein degradation based on protein half-lives. Scatter plot highlighting protein half-lives between CHX and CHX+ACBI2 (A) or CHX+**AuPROTAC** (B), including the associated rank-based plot to assess the magnitude of protein half-life difference (Δ*t*_1/2_). First-order decay fit of filamin A (FLNA, (C)) and metallothionein-1X (MT1X, (D)) on the experimental protein intensities in the different conditions. Positive Δ*t*_1/2_ indicates proteins whose half-life has been reduced due to PROTAC degradation.

### Limitations of the Method

Identifying protein targets by degradation requires their successful detection by proteomic methods. Here, we have verified the detection of proteins of interest for VHL and CRBN interacting PROTACs by deep proteome profiling in parallel to establishing the steady-state system. The CHX chase assay is based on global translation inhibition, which impacts cell viability. A maximum chase period of 8 h was selected as an appropriate compromise to determine protein half-lives in conjunction with a negligible cytotoxic effect. Consequently, the presented method can be effectively used to determine protein half-lives <50 h. Additionally, blocking protein synthesis is appropriate to enhance degradation dynamics induced by PROTACs; however, it may overestimate the efficiency of protein degradation due to a lack of cellular compensation reactions. Characterizing degradomes of PROTACs in the presented experimental setup via differences in protein half-lives requires the robust determination of protein half-lives under two conditions, i.e., CHX and CHX+PROTAC, to calculate the magnitude of protein half-life reduction. This is limited to proteins for which proteins half-lives can be calculated in these two conditions. Finally, it is worth noting that cellular uptake/excretion kinetics influence protein half-life dynamics, potentially biasing the initial assumption of first-order decay kinetics for determining protein half-life by introducing a lag time.

### Conclusions and Perspectives

Here, we report on the design and synthesis of the first gold-based PROTAC (**AuPROTAC**) featuring a unique mode of covalent binding to cysteine residues in proteins, relying on a two-step mechanism whereby the Au­(III) warhead first binds to thiolate groups and then templates the C–S bond-forming reaction. The reactivity of the compound with model cysteine residues was first confirmed by ^1^H NMR spectroscopy.

The degradation targets of **AuPROTAC** were then characterized in a nonproliferative cellular steady-state that maintains a static protein turnover. Employing a translation inhibitor (CHX) in this system efficiently decouples protein degradation from protein down-regulation and enables the discovery of protein degradation targets. Degraded protein targets are identified by differential analysis against a translation-inhibited control and subsequently verified by degradation kinetics. The systematic approach was confirmed by the selective degradation of SMARCA2 and PBRM1 of the optimized noncovalent PROTAC ACBI2.^46^ The **AuPROTAC** was then discovered to quantitatively degrade MERTK and TXNL1, and both were successfully rescued by proteasome inhibition. The degradomes were further quantified by the PROTAC-induced reduction of target protein half-lives. ACBI2 and **AuPROTAC** affected the protein half-lives of 221 and 95 unique proteins, respectively. Further experiments in relevant disease models will be conducted to characterize the effects of **AuPROTAC** degradation on MERTK and TXNL1. Overall, the applied translation-inhibition chase assay can be used to efficiently explore degradomes of CRBN and VHL recruiter-based PROTACs and reveals detailed degradation dynamics. It complements a recently published approach for the selective analysis of protein degradation by mass spectrometry at proteomic scale,^47^ and can be implemented in PROTAC discovery and validation campaigns.^53^

## Experimental Section

### Materials and Methods

Solvents and reagents (reagent grade) were all commercially available and used without further purification. Reactions involving gold were carried out protected from light. Flash column chromatography was performed on silica gel 60A (particle size 40–63 μm). Thin-layer chromatography (TLC) was performed using Merck Millipore silica gel 60 F-254 plates and analyzed with UV light. ^1^H and ^13^C­{^1^H} NMR spectra were recorded on a Bruker AV400 Ultrashield, Bruker 500HD or Bruker AV500 spectrometer with a 5 mm QNP cryoprobe. Chemical shifts δ are reported in parts per million (ppm) and coupling constants *J* are reported in Hertz (Hz), with residual ^1^H and ^13^C signals corresponding to the deuterated solvents as internal standards. Elemental Analysis (EA) for C, H, and N was performed by the microanalytical laboratory at the Technical University of Munich. High-resolution electrospray ionization mass spectrometry (HR-ESI-MS) was carried out on a Thermo Fisher Exactive Orbitrap mass spectrometer equipped with a Thermo Fisher ESI source. Samples were prepared in acetonitrile and syringe filtered before direct injection; ions were detected in positive mode. HR-Desorption­(D)­ESI-MS experiments were performed on a Thermo Fisher Q Exactive Plus mass spectrometer operated in positive ion mode. Data were obtained with a nominal mass resolution of 70,000 Da within the mass to charge (*m*/*z*) range of 700–1000 for **AuPROTAC**. The capillary temperature was set to 320 °C, the S-Lens RF value to 100 and the maximum injection time to 250 ms. A mixture of HPLC grade methanol and water (95:5, v/v) was used as the electrospray solvent at a flow rate of 1.50 μL/min and a spray voltage of 4.5 kV. The nebulizing gas pressure was set to 10 bar (Nitrogen N5.0). The DESI geometrical parameters were as follows: a sprayer-to-sample distance of 1.5 mm, a sprayer-to-inlet distance of 6 mm, a spray angle of 75°, and a collection angle of 10°. Compounds **1** (*tert*-butyl (2,6-dioxopiperidin-3-yl)­carbamate), **2** ^50^ (2-(2,6-dioxopiperidin-3-yl)-4-fluoroisoindoline-1,3-dione) and **Au^oxym^** ^51^ were synthesized according to literature procedures.

### Synthesis and Characterization

#### Synthesis of *tert*-Butyl (2,6-Dioxopiperidin-3-yl)­carbamate (**1**) and 2-(2,6-Dioxopiperidin-3-yl)-4-fluoroisoindoline-1,3-dione (**2**)


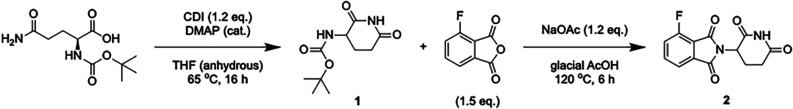


#### Synthesis of *tert*-Butyl (2-(4-(2-(2,6-Dioxopiperidin-3-yl)-1,3-dioxoisoindolin-4-yl)­piperazin-1-yl)­ethyl)­carbamate (**3**)


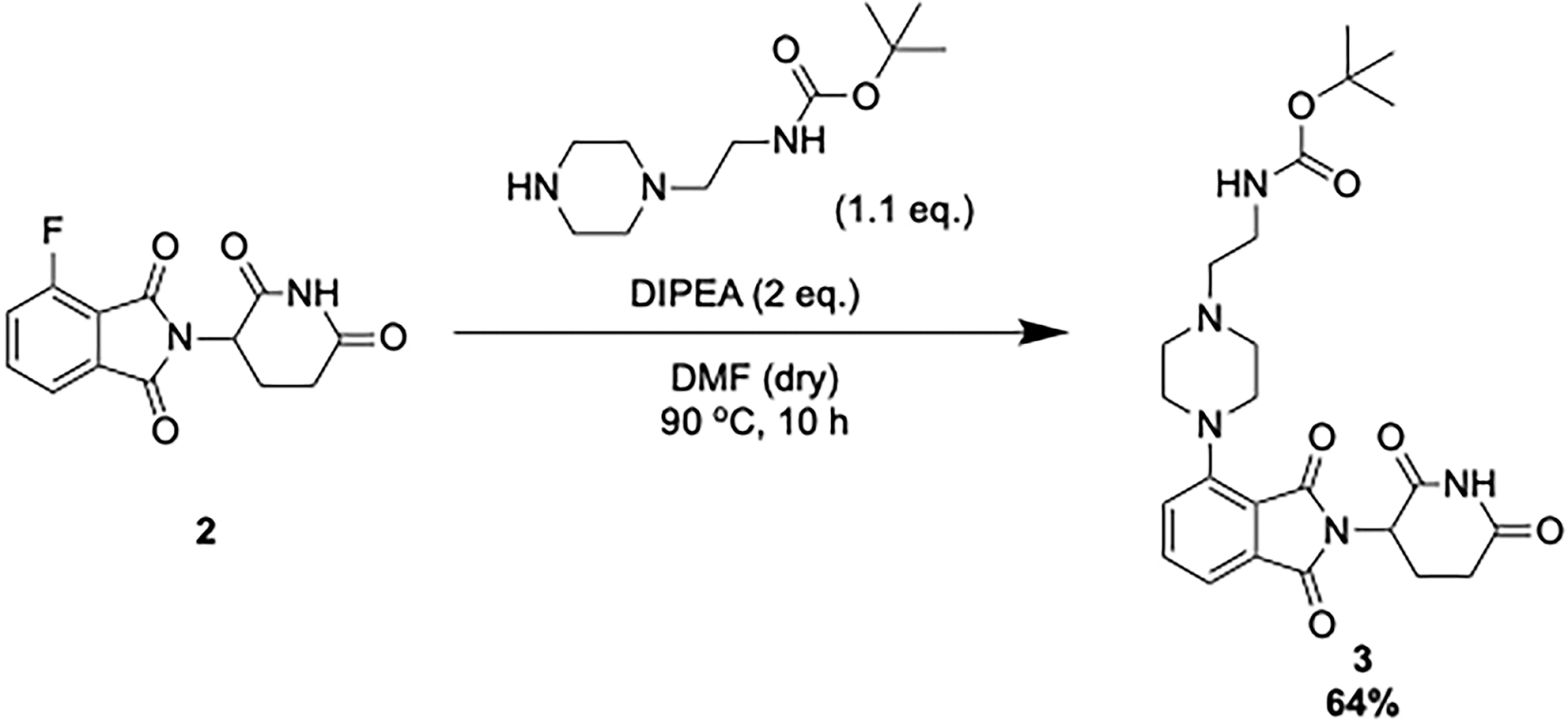
To a mixture of the *tert*-butyl (2-(piperazin-1-yl)­ethyl)­carbamate (0.9114 g, 3.97 mmol, 1.1 equiv) and 2-(2,6-dioxopiperidin-3-yl)-4-fluoroisoindoline-1,3-dione (**2**, 0.888 g, 3.21 mmol, 1 equiv) under nitrogen conditions in dry dimethylformamide (DMF, 25 mL), *N,N*-Diisopropylethylamine (DIPEA, 1.12 mL, 6.43 mmol, 2 equiv) was added under stirring. The solution was left to stir for 18 h at 90 °C, before the reaction mixture was cooled to RT. Upon cooling, H_2_O (200 mL) was added, and the solution was extracted with ethyl acetate (EtOAc, 3 × 100 mL). The combined organic layers were then washed with H_2_O (100 mL) and brine (100 mL), dried over magnesium sulfate (MgSO_4_), and filtered. The solvent was then removed under reduced pressure, before purification of the crude product by flash column chromatography (gradient of *n*-hexane/EtOAc 1:1 to 100% EtOAc, product eluted at 100% EtOAc). The purified product was then collected, and the solvent was removed under reduced pressure. To remove the DIPEA salt, the product was dissolved in acetone and Amberlite FPA66 anion exchange resin beads were added to the product solution. This was then left to stir for 30 min before filtration and subsequent removal of solvent to obtain the clean product **3** (1.0064 g, 2.07 mmol, 64%).

^**1**^ **H NMR** (400 MHz, Acetone-*d*_6_) δ 9.85 (s, 1H, H*a*), 7.70 (t, *J* = 7.8 Hz, 1H, H*b*), 7.34 (dd, *J* = 7.8, 4.9 Hz, 2H, H*c*), 5.78 (s, 1H, H*k*), 5.11 (dd, *J* = 12.6, 5.4 Hz, 1H, H*d*), 3.38 (t, *J* = 4.9 Hz, 4H, H*g*), 3.24 (q, *J* = 6.2 Hz, 2H, H*j*), 3.03–2.89 (m, 1H, H*f*), 2.76 (s, 2H, H*f*, H*e*), 2.67 (t, *J* = 4.9 Hz, 4H, H*h*), 2.52 (t, *J* = 6.4 Hz, 2H, H*i*), 2.25–2.16 (m, 1H, H*e*), 1.41 (s, 9H, H*l*).

^**13**^ **C NMR** (101 MHz, Acetone-*d*_6_) δ 172.62 (C1), 170.14 (C2), 168.06 (C3), 167.46 (C4), 151.17 (C5), 136.46 (C6), 135.31 (C7), 124.24 (C8), 118.32 (C9), 115.58 (C10), 78.54 (C11), 58.46 (C12), 53.83 (C13), 51.80 (C14), 50.16 (C15), 38.33 (C16), 32.02 (C17), 28.67 (C18), 23.35 (C19).

**HR-ESI-MS** (CH_3_CN, pos. mode): C_24_H_32_N_5_O_6_^+^ (**3**): exp. 486.2309 (calc. 486.2346). Mass error: −7.609 ppm.

#### Synthesis of 4-(4-(2-Aminoethyl)­piperazin-1-yl)-2-(2,6-dioxopiperidin-3-yl)­isoindoline-1,3-dione (**4**)


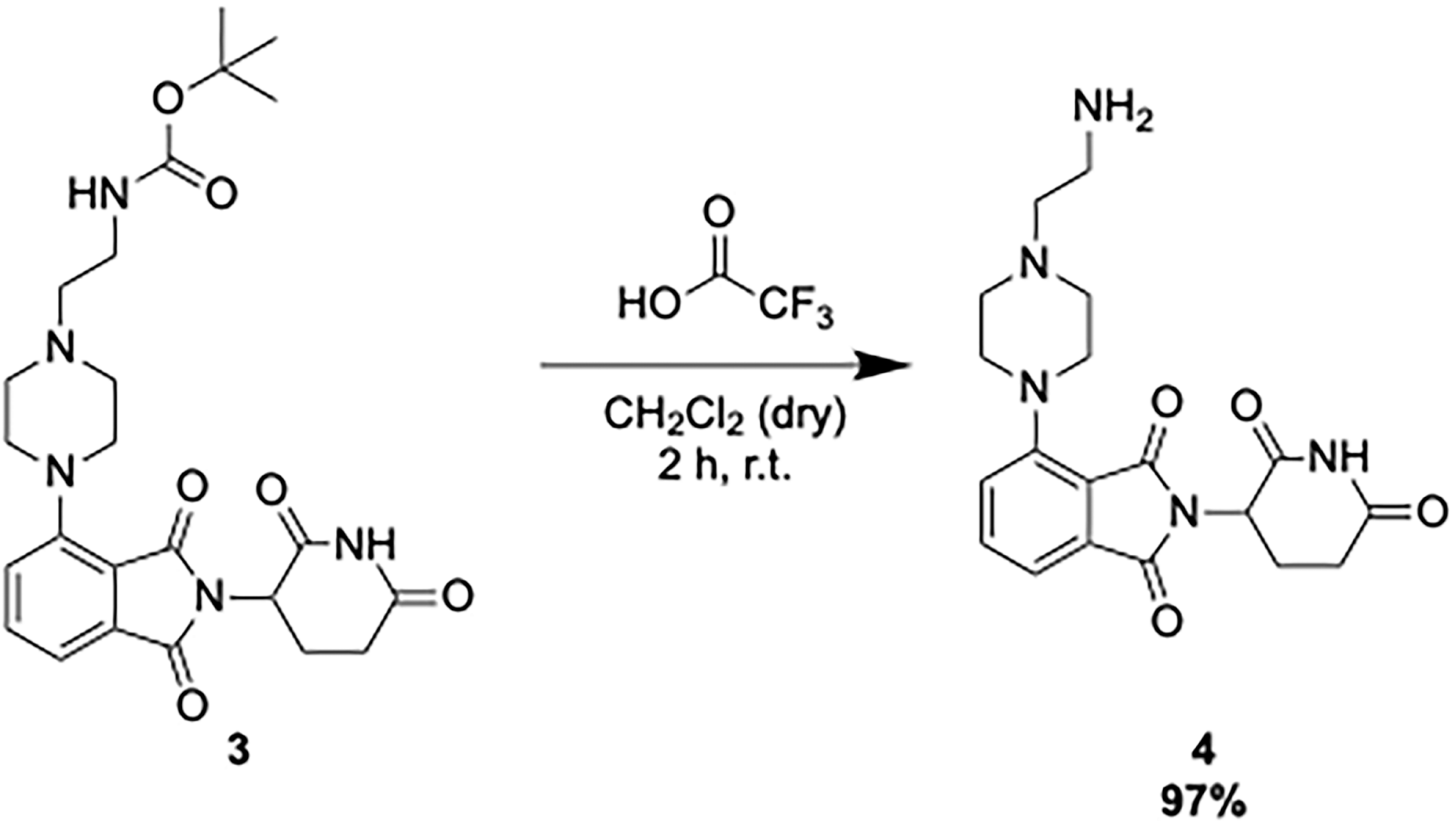
For the BOC-deprotection, *tert*-butyl (2-(4-(2-(2,6-dioxopiperidin-3-yl)-1,3-dioxoisoindolin-4-yl)­piperazin-1-yl)­ethyl)­carbamate (**3**, 0.2 g, 0.41 mmol) was dissolved in dry dichloromethane (DCM, 10 mL) before the addition of trifluoracetic acid (10 mL). The mixture was then stirred for 2 h at RT. To remove excess acid, Amberlyst A-21 resin beads were added to the mixture and this was left to stir for 30 min. The resin beads were then removed by filtration and washed with 1:1 DCM/methanol (MeOH, 10 mL). The solvent was then removed under reduced vacuum and the product was washed with *n*-hexane (20 mL) to yield **4** (0.1537 g, 0.40 mmol, 97%).

^**1**^ **H NMR** (400 MHz, Acetone-*d*_6_) δ 7.76 (t, *J* = 7.8 Hz, 1H, H*b*), 7.44 (dd, *J* = 14.2, 7.8 Hz, 2H, H*c*), 5.13 (dd, *J* = 12.5, 5.6 Hz, 1H, H*d*), 4.47 (t, *J* = 6.2 Hz, 2H, H*j*), 3.89 (t, *J* = 6.2 Hz, 2H, H*i*), 3.69 (d, *J* = 26.5 Hz, 8H, H*g*, H*h*), 3.05–2.89 (m, 1H, H*f*), 2.88–2.68 (m, 2H, H*f*, H*e*), 2.21 (dt, *J* = 10.4, 5.0 Hz, 1H, H*e*).

^**13**^ **C NMR** (101 MHz, Acetone-*d*_6_) δ 172.56 (C1), 167.93 (C2), 167.62 (C3), 150.01 (C4), 136.75 (C5), 135.17 (C6), 124.44 (C7), 119.05 (C8), 116.57 (C9), 53.94 (C10), 53.30 (C11), 50.19 (C12), 49.75 (C13), 37.26 (C14), 31.95 (C15), 23.33 (C16).

**HR-ESI-MS** (CH_3_CN, pos. mode): C_19_H_24_N_5_O_4_^+^ (4): exp. 386.1823 (calc. 386.1821). Mass error: 0.5179 ppm.

### Synthesis of AuPROTAC

In a Schlenk flask under argon, 4-(4-(2-aminoethyl)­piperazin-1-yl)-2-(2,6-dioxopiperidin-3-yl)­isoindoline-1,3-dione (**4**, 46.0 mg, 0.12 mmol, 1.25 equiv) was dissolved in dry DMF (2 mL). While stirring, 1-Hydroxybenzotriazole hydrate (HOBt·*n*H_2_O, 38.7 mg, 0.29 mmol, 3.0 equiv), *N*-(3-(Dimethylamino)­propyl)-*N*′-ethylcarbodiimide hydrochloride (EDC·HCl, 55.0 mg, 0.29 mmol, 3.0 equiv), **Au** ^**oxym**^ (50 mg, 0.09 mmol, 1.0 equiv) and DIPEA (20 μL, 0.11 mmol, 1.21 equiv) were added. The flask was washed with additional dry DMF (2 mL) and the yellow reaction solution was stirred vigorously under light exclusion for 18 h. To work up, the reaction solution was diluted with DCM (15 mL) and extracted with a 5% lithium chloride (LiCl) solution (1 × 25 mL). The aqueous phase was then extracted with DCM (1 × 10 mL) and the combined organic phases were extracted with brine (3 × 15 mL), dried over sodium sulfate (Na_2_SO_4_), filtered and concentrated under reduced pressure. The yellow crude product was then dissolved in DCM (4 mL) and precipitated with diethyl ether (Et_2_O, 30 mL × 4). After centrifugation (4000 rpm, 20 min), the solid precipitate was washed with *n*-hexane (30 mL), centrifuged (4000 rpm, 20 min) again and then dried in air, yielding the pure product as a yellow powder (20 mg, 0.022 mmol, 24%).

^**1**^ **H NMR** (400 MHz, Acetone-*d*_6_) δ 9.92 (s, 1H, H*a*), 9.50 (d, *J* = 6.0 Hz, 1H, H*b*), 8.91 (d, *J* = 8.0 Hz, 1H, H*c*), 8.49 (t, *J* = 7.8 Hz, 1H, H*d*), 7.95 (dd, *J* = 7.8, 5.0 Hz, 1H, H*e*), 7.73 (dt, *J* = 8.3, 6.7 Hz, 1H, H*f*), 7.65–7.57 (m, 1H, H*g*), 7.53 (d, *J* = 7.6 Hz, 1H, H*h*), 7.42 (t, *J* = 6.5 Hz, 2H, H*i*), 7.33 (dd, *J* = 13.1, 7.7 Hz, 2H, H*j*), 7.29–7.21 (m, 1H, H*k*), 5.13 (dd, *J* = 12.4, 5.4 Hz, 1H, H*l*), 4.84 (d, *J* = 5.0 Hz, 2H, H*m*), 3.33–3.26 (m, 2H, H*n*), 3.01–2.96 (m, 4H, H*o*), 2.83–2.67 (m, 8H, H*p*), 2.21 (dt, *J* = 4.4, 2.2 Hz, 2H, H*q*).

^**13**^ **C NMR** (126 MHz, Acetone-*d*_6_) δ 172.67 (1), 170.16 (2), 169.38 (3), 167.91 (4), 167.54 (5), 154.83 (6), 153.39 (7), 145.38 (8), 143.63 (9), 139.15 (10), 136.62 (11), 135.03 (12), 134.18 (13), 130.66 (14), 129.61 (15), 129.17 (16), 128.32 (17), 124.62 (18), 120.10 (19), 118.90 (20), 110.20 (21), 74.88 (22), 53.60 (23), 50.12 (24), 31.95 (25), 23.29 (26), 22.85 (27).

**HR-DESI-MS** (CH_3_CN, pos. mode): C_33_H_32_AuCl_2_N_7_O_6_Na^+^ (**AuPROTAC**): exp. 912.1317 (calc. 912.1346), mass error = −3.1794 ppm.

**EA.** Calculated for C_33_H_34_AuCl_2_N_7_O_7_ (**AuPROTAC**+H_2_O) [%] C, 43.63; H, 3.77; N, 10.79. Found [%]: C, 43.94; H, 3.68; N, 10.40.

### Stability Studies and Reactivity with *N*-Acetylcysteine (NAC)

**AuPROTAC** (5 μmol, 1 equiv) was tested for its stability at RT by ^1^H NMR spectroscopy in a solvent mixture of DMSO-*d*_6_ and D_2_O (9:1, 500 μL). Spectra were recorded over time at time 0, 5 min, 20 min, 1, 6, 8, and 24 h. Subsequently, the ability of **AuPROTAC** to arylate the cysteine of NAC was investigated in the same conditions but following the addition of NAC (15 μmol, 3.0 equiv).

### Cell Culture

The acute myeloid leukemia cell line HL-60 (AML FAB-M2) was kindly provided by M. Jakupec from the Faculty of Chemistry, University of Vienna, Austria. HL-60 cells were cultured in suspension T75 flasks with ventilated caps (SARSTEDT) using RPMI-1640 medium with sodium bicarbonate (Gibco) containing 10% heat-inactivated FBS (Gibco) and 1% Penicillin-Streptomycin solution (from 100×, Sigma-Aldrich). All cell culture procedures were performed in a HERASAFE KS laminar flow cabinet (Thermo Fisher Scientific) and cells were incubated in a HERACELL 150i CO_2_ incubator (Thermo Fisher Scientific) at 37 °C in a humidified environment with 5% CO_2_.

Cell counting was performed by mixing 50 μL of cell suspension with equal volume of 0.4% Trypan Blue solution (Sigma-Aldrich). An aliquot of 10 μL of the resulting solution was then pipetted on the clean sample slide of a Brightfield Cell Counter (DeNovix CellDrop BF). A dedicated method was used, accounting for the different diameters, dilution factors as well as cell roundness. Exposure and focus pane of the device were checked prior to each cell count and adapted when necessary.

Stock solutions of the compounds used throughout the experiments were freshly prepared in DMSO, including **AuPROTAC** (11 mM) and ACBI2 (20 mM). Cycloheximide (CHX, 60 mM) was dissolved in ethanol, and phorbol 12-myristate 13-acetate (PMA, 1 mg·mL^–1^) was dissolved in DMSO. The compounds were further diluted to the appropriate concentrations in complete medium and the DMSO concentration did not exceed 0.5%. Differentiation of HL-60 cells (500,000 cells) was performed in 6-well plates (SARSTEDT) and induced by PMA (100 or 500 ng·mL^–1^) over 72 h.

### Viability Assays

The colorimetric MTT (3-(4,5-dimethylthiazol-2-yl)-2,5-diphenyltetrazolium bromide; 97.5%, Sigma-Aldrich) assay was employed to assess cell viability. The MTT assays were carried out in standard flat-bottom 96-wellplates (SARSTEDT) using 10,000 HL-60 cells per well and initially treated with PMA (100 ng·mL^–1^) over 72 h. Afterward, the differentiated HL-60 cells were treated with either cycloheximide (0.1 nM–100 μM), **AuPROTAC** (0.5 nM–50 μM) or ACBI2 (0.05 nM–50 μM) for 24 h. Then, MTT reagent (20 μL of 5 mg·mL^–1^) was added and incubated for an additional 4 h. The solution was removed, and the remaining crystals were dissolved in DMSO (50 μL). The absorbance of each well was measured at 570 nm in a Multiskan GO photometric plate reader controlled via SkanIt Software (v.3.2.0.35 Research Edition, both from Thermo Fisher Scientific) from a desktop computer. The averaged blank readout was subtracted from all absorbance values, and the average absorbance of the control wells was then used to normalize the remaining results to the untreated cells, yielding the cell viability. Each experiment was performed in three independent replicates using each at least three technical replicates.

### Cell Proliferation Assessment upon PROTACs Treatment

The steady state of differentiated HL-60 cells upon treatment with the PROTACs was assessed steady state of differentiated HL-60 cells upon treatment with the PROTACs was assessed a Cellwatcher M (PHIO scientific GmbH). One 6-well plate was prepared by seeding HL-60 cells at a density of 500,000 cells per well in 1.8 mL complete RPMI1460 medium containing 500 ng·mL^–1^ PMA. The 6-well plate was placed in the Cellwatcher module in the incubator at 30 °C, where differentiation was allowed to proceed for 72 h under constant monitoring of the area of adherent cells. Then, the assay was paused and the samples treated in duplicates to achieve final in-well concentrations of 10 μM CHX and 1 nM ACBI2, or 10 μM CHX and 60 μM **AuPROTAC** as well as the corresponding solvent control condition. The samples were then observed for further 24 h.

### Nucleocytoplasmic Fractionation for Proteome Profiling

Proteomic profiling to assess the presence of key proteins for PROTAC activity were performed with HL-60 cells. Six replicates were prepared by seeding 2·10^6^ cells per T25 culture flasks (SARSTEDT) in 5 mL complete medium. The cells were differentiated with PMA (100 ng·mL^–1^) for 72 h. A nucleocytoplasmic fractionation was then performed, working on ice throughout the protocol. First, the medium in T25 culture flasks was removed and the cells were washed twice with 5 mL cold PBS. Then, isotonic fractionation buffer (1 mL) was added, which contains 10 mM HEPES pH 7.4, 10 mM NaCl, 3.5 mM MgCl_2_, 1 mM EGTA, 250 mM sucrose, 0.5% triton x-100, 1% PIC and 1% PMSF, and the cells were then scraped from the bottom of the flask using a cell scraper. The cell suspension was transferred to a labeled 15 mL Falcon tube using a 23G needle attached to a 1 mL syringe. The membrane was lysed by applying shear stress using the 23G needle. Complete lysis was monitored under a light microscope. The suspension was centrifuged (3500 rpm, 5 min) at 4 °C. The supernatant containing the cytoplasmic fraction was precipitated in ice cold EtOH and stored at −20 °C. The nuclear pellet was dried, solubilized with TE-NaCl solution (100 μL, 10 mM Tris-HCl, 1 mM EDTA, 500 mM NaCl) and incubated for 10 min on ice. Then, TE-TritonX solution (900 μL, 10 mM Tris-HCl, 1 mM EDTA, 0.5% Triton X-100) was added and incubated for another 15 min on ice. The sample tubes were then centrifuged (3500 rpm, 5 min) at 4 °C before being precipitated in ice-cold EtOH and stored at −20 °C. These samples were centrifuged (5000 rpm, 30 min) at 4 °C to pellet the proteins. The ethanolic supernatant was decanted and dried in a vacuum desiccold EtOH and stored at −20 °C. These samples were centrifuged (5000 rpm, 30 min) at 4 °C to pellet the proteins. The ethanolic supernatant was decanted and dried in a vacuum exicator. The cytoplasmic protein pellet was resuspended in 70 μL lysis buffer (8 mM urea, 50 mM TEAB, 0.2 mM SDS), while the nucleic fraction pellet was resuspended in 20 μL lysis buffer.

### Electrophoresis by SDS-PAGE

Cytoplasmic and nuclear fractions of differentiated HL-60 cells were loaded on a previously cast 12% Acrylamide gel. The sodium dodecyl sulfate polyacrylamide slab gel was cast in separate steps for discontinuous electrophoretic separation of the proteins in the sample. The separating gel was cast from a solution with final concentrations of 12% acrylamide/piperazine diacrylamide (PDA), 375 mM Tris-HCl (2 M, pH = 8.8), 0.1% sodium dodecyl sulfate (SDS), 0.075% tetramethylethylenediamine (TEMED) and 0.045% ammonium persulfate (APS) in water. The separation gel was allowed to polymerize for 40 min. The stacking gel consisted of a solution with final concentrations of 4% Acrylamide/PDA, 125 mM Tris-HCl (2 M, pH = 6.8), 0.1% SDS, 0.1% TEMED and 0.05% APS in water. A sample comb with ten spacers was then inserted and the gel left to polymerize for 1 h.

The gel was then placed into an SDS-PAGE apparatus (Mini PROTEAN Tetra Cell, Bio-Rad Laboratories Inc.) which was then filled with Tris-Glycin buffer. The molecular weight marker (5 μL, Precision Plus Protein Dual Color Standards, Bio-Rad Laboratories Inc.) was pipetted into one lane. Subsequently 5× SDS sample buffer (5 μL) was pipetted into every other lane. Both protein samples of nucleocytoplasmic fractions (62.4 μg) were loaded into separate lanes of the gel and topped up to a volume of 25 μL with sample buffer. The remaining lanes were filled with 25 μL of sample buffer and used as blanks. A maximum current of 40 mA and a maximum voltage of 250 V was set on the control unit (PowerPac Universal, Bio-Rad Laboratories Inc.) before starting the electrophoresis. The SDS-PAGE was run until a separation of the samples over a distance of 1.5 cm was achieved. The gel was then removed from the apparatus and placed in fixing solution (50% Methanol, 10% acetic acid in water) for 30 min. The following steps were performed at RT while slowly shaking the gel on a plate mixer. The gel was washed for 10 min in 50% Methanol followed by two 5 min steps in water. Sensitization was performed by transferring the gel into a 0.02% sodium thiosulfate solution for 1 min, which was then followed by two brief rinsing steps in water. The gel was then stained in a 0.1% silver nitrate solution over the course of 10 min, briefly rinsed with water and developed in a 3% sodium carbonate, 0.05% formaldehyde solution over the course of 15 s. The gel was again rinsed in water and then placed into a 1% acetic acid solution until further processing.

The lanes containing the cytoplasmic and nuclear fractions were cut from the rest of the gel. The gel pieces were then horizontally cut into eight bands of similar sizes, which contained proteins with cascading molecular weights. Each of the bands was then vertically cut four times; the pieces belonging to one band were then transferred to a labeled Eppendorf tube.

The gel pieces inside each Eppendorf tube were then destained in 200 μL of a 15 mM potassium ferricyanide (K_3_[Fe­(CN)_6_]), 50 mM sodium thiosulfate solution by vortexing the tubes until the gel pieces were transparent. The supernatant was discarded and the gel pieces were then washed four times in total by shaking the tubes for 10 min and 1200 rpm at RT. The first washing step was in 400 μL ammonium bicarbonate (ABC, 25 mM) solution, followed by 400 μL acetonitrile. These two steps were then repeated and the protein samples were dried in a vacuum concentrator. The dried pellets were dissolved in 30 μL sample buffer (7.5 mM urea, 1.5 mM thiourea, 65 mM CHAPS, 0.05 mM SDS and 100 mM DTT) and stored at −20 °C until further processing.

### Optimization of Cycloheximide (CHX) Concentration for Global Translation Inhibition

In 6-well plates, HL-60 cells (500,000 cells well^–1^) in complete RPMI1640 culture medium (1.8 mL) were differentiated with PMA (100 or 500 ng·mL^–1^) for 72 h. Then, CHX was added in 200 μL complete medium to achieve final concentrations of 40 nM, 200 nM, 1 μM and 10 μM, and the cells were incubated for 24 h. Each condition was run in triplicates. The medium was removed and the wells were washed twice with cold PBS (2 mL). The cells were scraped from the bottom of the wells twice using 60 μL and then 30 μL of sodium deoxycholate lysis buffer (SDC, 102 mM in Tris-HCl 100 mM, pH 8.5). These aliquots were combined in a labeled Eppendorf tube and heated to 95 °C (5 min, 1400 rpm) in a preheated Thermomixer Comfort (Eppendorf AG). Samples were then stored at −20 °C until further processing.

### Cycloheximide Chase Assay

HL-60 cells were and seeded into 6-wells at a density of 500,000 cells per well in completed culture medium (1.8 mL) containing PMA (500 ng·mL^–1^) and differentiated over the course of 72 h. Cells were then treated with complete culture medium (200 μL) containing either solvent vehicle control (DMSO or EtOH), CHX (10 μM), ACBI2 (1 nM + 10 μM CHX) or **AuPROTAC** (60 μM + 10 μM CHX) in triplicates. Whole cell lysates were obtained after 0.5, 1, 2, 4, and 8 h incubation. Control cells were additionally harvested directly at the beginning of the experiment (*t* = 0). Whole-cell lysates were generated by removing medium and washing the wells twice with cold PBS (2 mL). The cells were scraped from the bottom of the wells twice using 60 μL and then 30 μL of SDC buffer, similarly, as described above. These aliquots were combined in a labeled Eppendorf tube and heated to 95 °C (5 min, 1400 rpm) in a preheated Thermomixer Comfort (Eppendorf AG). Samples were then stored at −20 °C until further processing.

### Rescue of Protein Degradation by Proteasome Inhibition

Bortezomib (25 nM final concentration) was added to PMA-differentiated HL-60 cells (from 500,000 cells, 72 h) in 6-wells using triplicates per planned condition. The plate was subsequently incubated for 4 h before the addition of ACBI2 (1 nM final concentration) or AuPROTAC (60 μM final concentration). The DMSO content of the treatments was matched among the conditions. The plate was then incubated for further 4 h before being worked up. Whole cell lysates were generated, as described above, by removing medium and washing the wells twice with cold PBS (2 mL). The cells were scraped from the bottom of the wells twice using 60 μL and then 30 μL of SDC buffer. These aliquots were combined in a labeled Eppendorf tube and heated to 95 °C (5 min, 1400 rpm) in a preheated Thermomixer Comfort (Eppendorf AG). Samples were then stored at −20 °C until further processing.

### Assumptions to Use the Cycloheximide Chase Assay to Study PROTAC-Induced Protein Degradation

Protein turnover is characterized by a logistic equation of protein synthesis and degradation. Protein synthesis is a zero-order reaction, where he protein synthesis rate *k*_syn_ can be calculated as a function of the incremental increase of a protein LFQ-intensity (PROT_LFQ_) over a given time difference (d*t*). In contrast, protein degradation is assumed to underly first-order kinetics, therefore the protein degradation rate *k*_deg_ also depends on the initial LFQ-intensity (PROT_LFQ_)_0_ (1).

1 $$\frac{d\left({\mathrm{Prot}}_{\mathrm{LFQ}}\right)}{dt}=k_{\mathrm{syn}}-{\mathrm{Prot}}_{\mathrm{LFQ}}\cdot k_{\deg},\mathrm{with},{\mathrm{Prot}}_{\mathrm{LFQ}}\left(t\right)={\left({\mathrm{Prot}}_{\mathrm{LFQ}}\right)}_{0}\cdot e^{-k_{\deg}t}$$

Under steady-state conditions, protein synthesis and degradation rates are constant, as are the protein intensities (2).

2 $$\frac{d\left({\mathrm{Prot}}_{\mathrm{LFQ}}\right)}{dt}=k_{\mathrm{syn}}-{\mathrm{Prot}}_{\mathrm{LFQ}}\cdot k_{\deg}=0\to {\mathrm{Prot}}_{\mathrm{LFQ}}=\frac{k_{\mathrm{syn}}}{k_{\deg}}$$

Blocking protein synthesis by a translation inhibitor would further eliminate *k*_syn_ and protein half-lives (*t*_1/2_) are governed by first-order decay kinetics (3). In this case, protein half-lives can be calculated from the slope of the ln­(LFQ intensity) over time

3 $$t_{1/2}=\frac{\ln\left(2\right)}{k_{\deg}}$$

### Proteolytic Digestion

Protein quantification in sample lysates was performed by means of the Bicinchoninic acid (BCA) colorimetric assay and samples were adjusted to 20 μg protein. Samples from nucleocytoplasmic fractionation were digested using ProtiFi or in-gel digestion protocols, while whole cell lysates were digested according to an in-solution protocol using StageTip desalting.

#### ProtiFi Digestion

Tubes containing the protein sample were topped up to 50 μL with lysis buffer before the addition dithiothreitol (DTT, 64 mM). The tubes were briefly vortexed and placed in a thermoshaker (95 °C, 300 rpm) for 10 min. After reaching RT, iodoacetamide (IAA, 12.5 μL, 486 mM) was added and the reaction tubes were incubated in a thermoshaker (30 °C, 300 rpm) for 30 min. After reaching RT, the samples were centrifuged (13,000*g*, 8 min). Thereafter, phosphoric acid (11.25 μL, 12%) was added to each sample, followed by S-trap buffer (866 μL), containing 90% MeOH and 10% TEAB (1 M). The solution of each sample was quantitatively transferred to the corresponding S-Trap spin columns (C02 mini, ProtiFi LLC). The column was centrifuged (1000 g, 1 min). The column was washed four times by adding S-Trap buffer (400 μL). The spin column was then placed on top of a fresh tube. The digestion enzyme mix was then prepared. MassSpec grade Trypsin Lys-C mix (PROMEGA GmbH) was diluted to 0.2 μg·μL^–1^ using the provided reconstitution buffer (PROMEGA GmbH) on ice. Typically, a mixture of digestion buffer (122.5 μL, 50 mM TEAB) and reconstituted enzyme (2.5 μL) per sample were added. The columns were then capped and placed inside an incubator (37 °C, 2 h). The digestion was performed at an enzyme to substrate ratio of 1:100. Then, digestion buffer (80 μL) were added to the column and centrifuged (1000*g*), followed by formic acid (FA, 80 μL, 0.2%). After centrifugation, 80 μL of 50% acetonitrile, 0.2% FA solution were added to the columns and the peptides were eluted into a fresh tube, and dried in a centrifugal vacuum concentrator (miVac DUO, Genevac). The dried samples were stored at −20 °C until further processing.

#### In-Gel Digestion

Protein samples of the cut bands were reduced with DTT (200 μL, 20 mM in 25 mM ABC) in a thermoshaker at 56 °C (1100 rpm, 30 min). The supernatant was discarded and the gel pieces washed twice with ABC followed by acetonitrile. Alkylation was performed by adding IAA (200 μL, 50 mM in 25 mM ABC) to the gel pieces and incubating in the dark in a thermoshaker at 37 °C (1100 rpm, 30 min). After reaching RT, the gel pieces were washed as previously described and dried to completion in the centrifugal concentrator (40 °C, 20 min). The digestion was performed by adding Trypsin/LysC in a 1:20 enzyme-to-substrate ratio to each band in 10 μL ABC (25 mM). After 15 min further 20 μL of ABC solution were added to the gel pieces. The samples were then incubated at 37 °C for 17 h. The samples were briefly centrifuged and cooled 4 °C. Trypsin/LysC was then added for a second digestion step in a 1:40 enzyme to protein ratio. The samples were then incubated at 37 °C for further 4 h. The supernatant containing the peptides from each tube was subsequently transferred to a correspondingly labeled sample tube. The peptides inside the gel pieces were extracted three times with 40 μL of ABC solution (25 mM) on a thermoshaker (1200 rpm, 15 min). The supernatant was transferred to the corresponding sample tube after each washing step. A further extraction step was performed with 5% formic acid, similarly to the previous steps. The peptide samples were then dried in the vacuum concentrator and stored at −20 °C until analysis.

#### In-Solution Digestion

Peptide samples were topped up to 90 μL with SDC lysis buffer. Subsequently, reduction/alkylation buffer (10 μL) was added and incubated on a thermoshaker (1400 rpm, 45 °C). The reduction/alkylation buffer contained TCEP (200 mM) and 2-chloroacetamide (800 μM) and the pH was adjusted to 7.5–8 using NaOH (5 M). After reaching RT, trypsin/Lys-C (1 μL, 0.2 μg·μL^–1^, enzyme-to-substrate ratio of 1:100) was added to each sample and incubated for 17 h on a thermoshaker (1400 rpm, 30 °C). Then, the samples were nearly dried in a vacuum concentrator (40 min, 40 °C). The StageTips were prepared by stacking two disks of a polystyrenedivinylbenzene-reversed phase sulfonate material (Empore 2241 SDB-RPS, 12 μm particle size, 47 mm; CDS Analytical LLC) into a pipette tip. A 100 μL volume of SDB-RPS loading buffer (99% IPA, 1% TFA) was added to each sample and was quantitatively transferred to the corresponding StageTip. The tips were then centrifuged (1500 g, 8 min) to allow the whole solution to pass through the RPS material. Then, loading buffer/wash buffer 1 (100 μL, 99% IPA, 1% TFA) and SDB-RPS wash buffer 2 (100 μL, 94.8% water, 5% ACN, 0.2% TFA) were sequentially added and centrifuged. The waste tube was then exchanged with a clean storage tube containing a glass LC-MS inlet. The peptides were directly eluted into the inlet using SDB-RPS elution buffer (60 μL, 39.8% water, 59.7% ACN, 0.5% NH_4_OH) followed by centrifugation (1500*g*, 5 min). The samples were finally dried in the vacuum concentrator (40 °C) and stored at −20 °C until analysis.

### Nanoflow Liquid Chromatography Tandem Mass Spectrometry (nLC-MS/MS)

Dried peptide samples were reconstituted in loading solvent (40 μL, 97.95% water, 2% acetonitrile, 0.05% TFA) and synthetic peptide standards (5 μL, 10 fmol·μL^–1^, in 30% FA) were added. Samples were briefly vortexed and centrifuged (10,000*g*, 5 min). The solution was quantitatively transferred to LC-MS glass vial inserts for analysis. Some samples of gel bands were additionally pooled after a first nLC-MS/MS run to evaluate pooling effects.

The chromatographic separation was performed on a Dionex UltiMate 3000 RSLCnano system (Thermo Fisher Scientific). The injection volume was 5 μL, which was loaded onto a precolumn (Acclaim PepMap C18 100, Thermo Fisher Scientific) using solvent A (99.9% water, 0.1% formic acid) at a flow rate of 10 μL·min^–1^. Peptides were separated on an Aurora emitter column (1.6 μm C18, 25 cm × 75 μm, IonOpticks) by applying a gradient ranging from 12% to 42% solvent B (79.9% acetonitrile, 20% water, 0.1% FA) over the course of 90 min at a flow rate of 300 nL·min^–1^. The applied method resulted in a total runtime of 140 min with inclusion of equilibration and washing phases. For the in-gel digested samples, an additional gradient of 41 min was applied starting from 12% up to 40% mobile phase B, which led to a total runtime of 85 min. Mass spectrometric analysis was performed on a timsTOF Pro (Bruker Daltonics) mass spectrometer running in parallel accumulation-serial fragmentation (PASEF) mode and data dependent acquisition (DDA). An *m*/*z* scan range of 100–1700 was set to acquire MS1 and MS2 spectra. An inverse reduced ion mobility (1/*k*0) scan ranging from 0.6–1.6 V·s·cm^2^ contributed to a total ramp time of 100 ms to achieve trapped ion mobility separation. The total cycle time was 1.16 s, including 10 PASEF MS/MS scans per cycle.

### Proteomic Data Processing

The acquired data were processed using MaxQuant (version 1.6.17.0) with the Andromeda search engine to enable identification and label-free quantification (LFQ) of proteins and searched against the SwissProt *Homo sapiens* database (14.12.2019 with 20380 canonical entries). False discovery rates for peptide-spectrum match (PSM) and protein were set to 0.01, the “match between runs” setting was enabled with a matching time window of 0.7 min and an alignment time window of 20 min. Further criteria included a MS/MS mass tolerance of 40 ppm and a maximum of two missed cleavages. A minimal requirement for protein identification was set to one unique peptide. Carbamidomethylation of cysteine was set as fixed modification while methionine oxidation and the acetylation of the protein N-terminus were included in the search as variable modifications. Perseus (version 1.6.14.0) was used for filtering and imputation. First, proteins only identified by site, common contaminants as well as proteins matching reversed sequences were filtered out. LFQ-values of remaining entries were log_2_-transformed.

Entries with missing values were then imputed with values from a normal distribution (downshift: 1.8, width: 0.3). Volcano plots were generated using a two-sided *t* tests with a *s*0 = 0.1, FDR = 0.05 and 250 permutations. Data matrices prior and after replacement of missing values were exported from Perseus and imported to Python (version 3.11.5) for further filtering steps, analysis and plotting.^77^ The Pandas package^78^ was used for the bulk of data processing, while the Matplotlib^79^ and Seaborn^80^ packages were employed for data plotting.

### Protein Fraction Remaining and Protein Half-Life Calculation

The protein fraction remaining (PFR) was calculated by normalizing the time-dependent LFQ-intensities (*t*_0.5h_ to *t*_8h_) to the averaged LFQ-intensity from CON (*t*_0h_) and expressed as percentage in each sample. Proteins were then analyzed based on their PFR over time. A strict filtering method was applied to the resulting data matrix to select for robust effects. First, the proteins had to be detected in three out of three replicates in all time points. For stable proteins, the PFR had to remain in an 80–120% boundary over all time points. The requirement for protein degradation patterns was a continuous intensity decrease across all the considered time points. Protein half-lives were then calculated from the slope of the ln­(LFQ value) over the different time points, which were found to obey a linear fit. This supports the validity of the assumption about first-order decay kinetics. This approach could be automated to obtain PFR decrease patterns and protein half-lives from the entire proteome data.

## Supplementary Material







## Data Availability

Proteomic data were submitted to the ProteomeXchange Consortium (https://proteomecentral.proteomexchange.org/) and are available in the PRIDE partner repository with identifier PXD067054. ## Reviewer access details for revision. Username: reviewer_pxd067054@ebi.ac.uk; Password: iTtb4QcuqFA5 ##.

## Notes

Characterization of compounds **3**, **4**, and **AuPROTAC** (Figures S1–S15); **AuPROTAC** stability study (Figure S16); data from the CHX+PROTAC chase assay (Figures S17–S18), protein half-lives determinations (Figure S19 and Tables S1–S2) (PDF)
Lists of identified proteins with degradation kinetics (Supporting Data 1) (XLSX)
Rankings of protein half-lives and half-life differences (Supporting Data 2) (XLSX)
