# Supplementary material for: A Gold-PROTAC Degrades the Oncogenic Tyrosine Kinase MERTK: Insights into the Degradome from a Steady-State System
Source: ACS Chem Biol. 2026 Jan 5;21(1):170–86. doi: 10.1021/acschembio.5c00860 (PMC12813982; doi:10.1021/acschembio.5c00860)
Supplement: Supplementary file 1 [file cb5c00860_si_001.pdf]

Supplementary Information

A Gold-PROTAC degrades the oncogenic tyrosine kinase MERTK: insights into the degradome from a steady-state system

Sophie R. Thomas,[a,b,‡] Thomas Iellici,[c,d,‡] Mihyun Park,[a] Elisabeth Klaus,[a] Andrea Bileck,[c,e] Christopher Gerner,[c,e] Samuel M. Meier-Menches,[b,c,e]* Angela Casini [a,]*

[a] *Chair of Medicinal and Bioinorganic Chemistry, Department of Chemistry, School of Natural Sciences, Technical University of Munich, Lichtenbergstr. 4, 85748 Garching, Germany.*

[b] *Institute of Inorganic Chemistry, University of Vienna, Waehringer Str. 42, 1090 Vienna, Austria.*

[c] *Institute of Analytical Chemistry, University of Vienna, Waehringer Str. 38, 1090 Vienna, Austria.*

[d] *Doctoral School in Chemistry, University of Vienna, Waehringer Str. 38, 1090 Vienna, Austria.*

[e] *Joint Metabolome Facility, Medical University of Vienna and University of Vienna, Waehringer Str. 38, 1090 Vienna, Austria.*

‡ These authors contributed equally

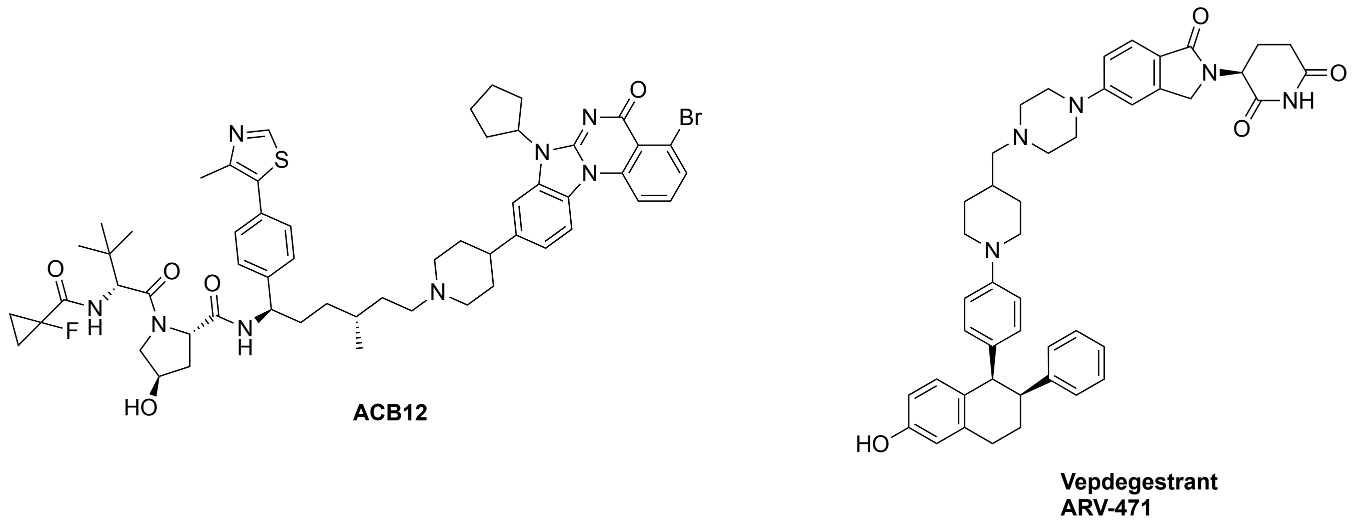

ACB12

Vepdegestrant
ARV-471

Scheme S1 – Structures of compounds ACB12 and ARV-471 discussed in this study.

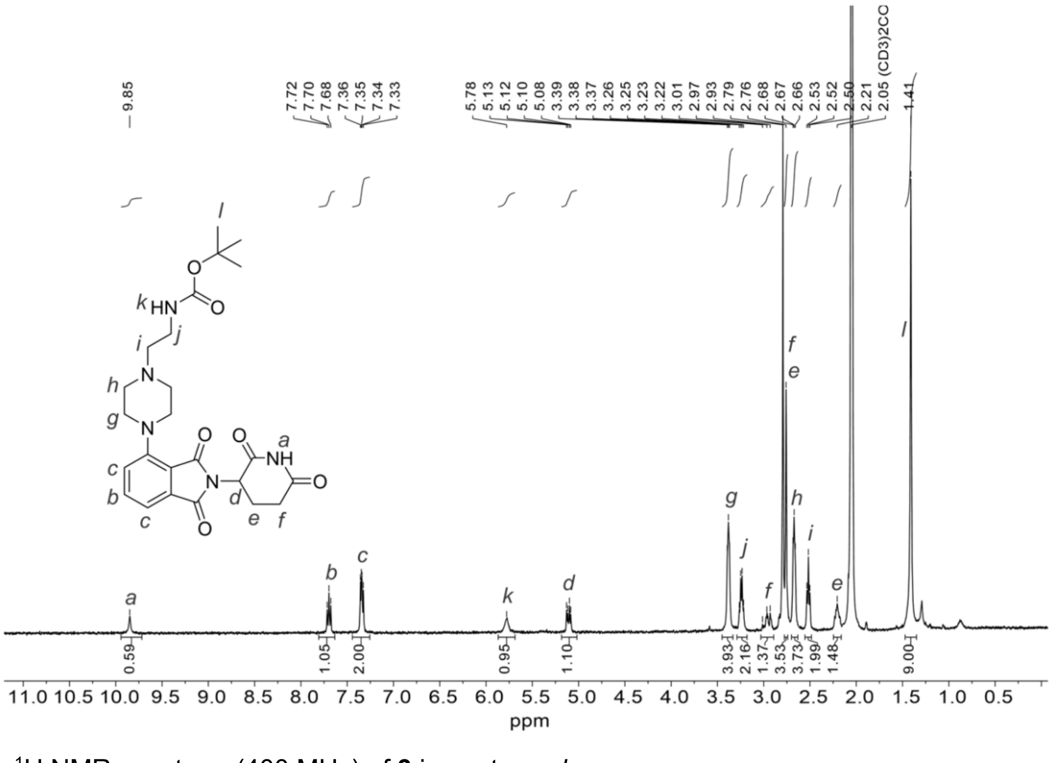

Figure S1. ^{1}H NMR spectrum (400 MHz) of **3** in acetone-d_6.

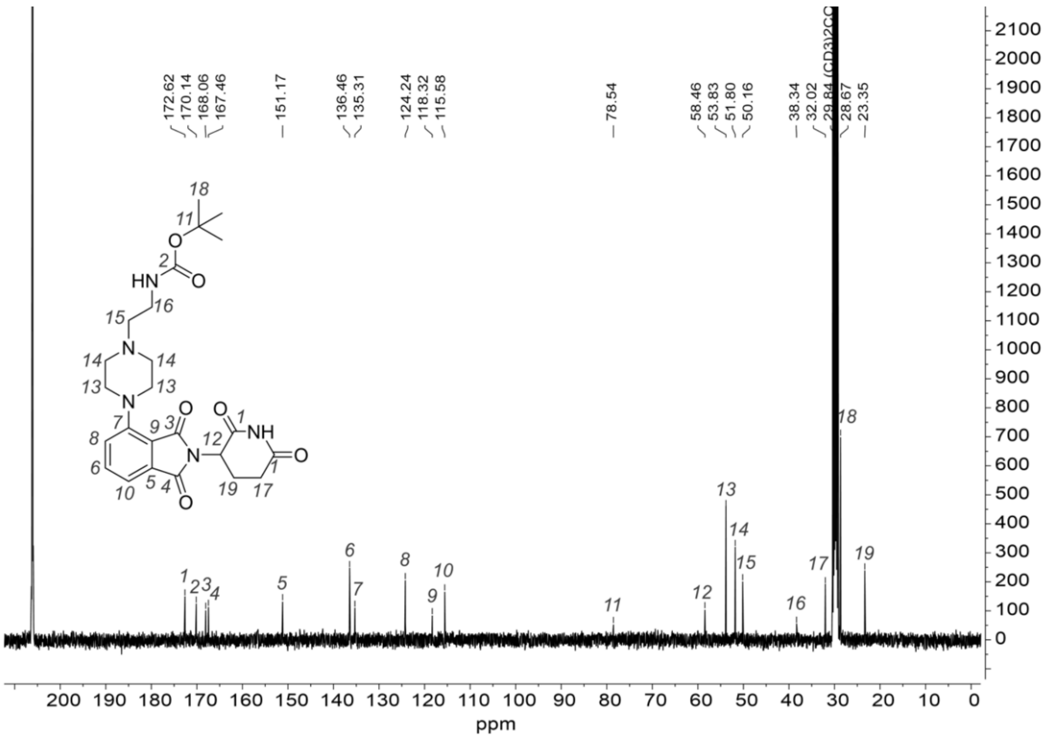

Figure S2. ^{13}C NMR spectrum (101 MHz) of **3** in acetone-d_6.

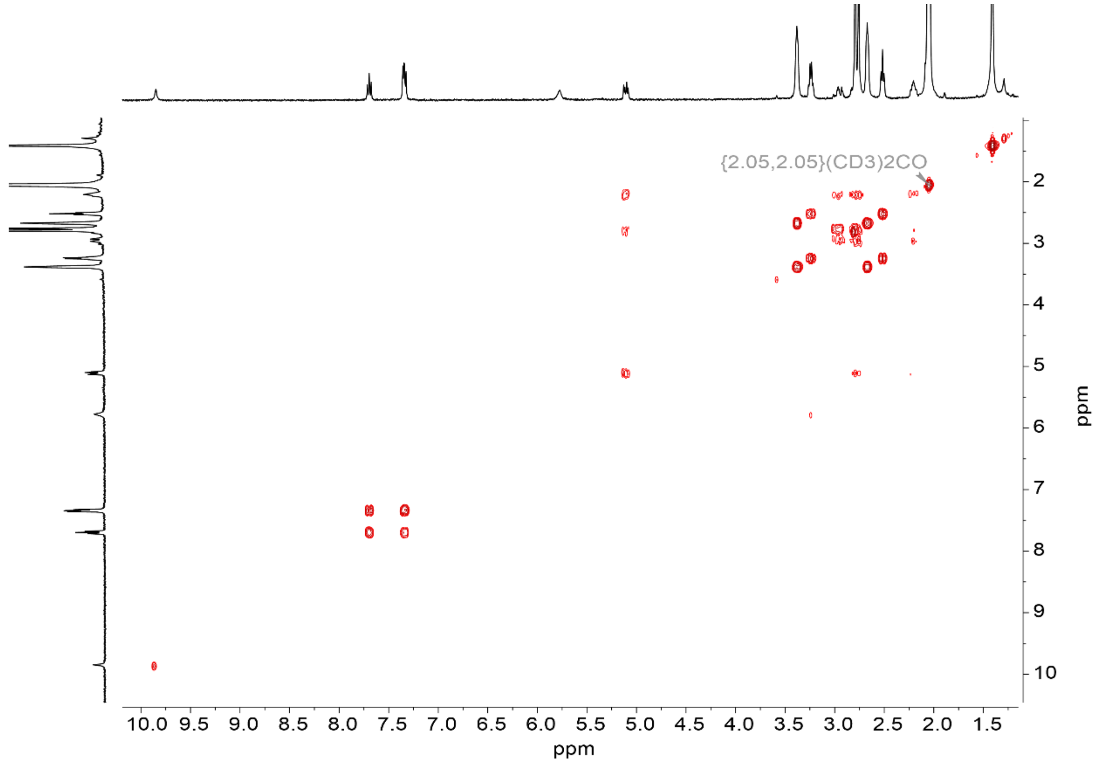

Figure S3. ^1H-^1H COSY NMR spectrum of **3** in acetone-d_6.

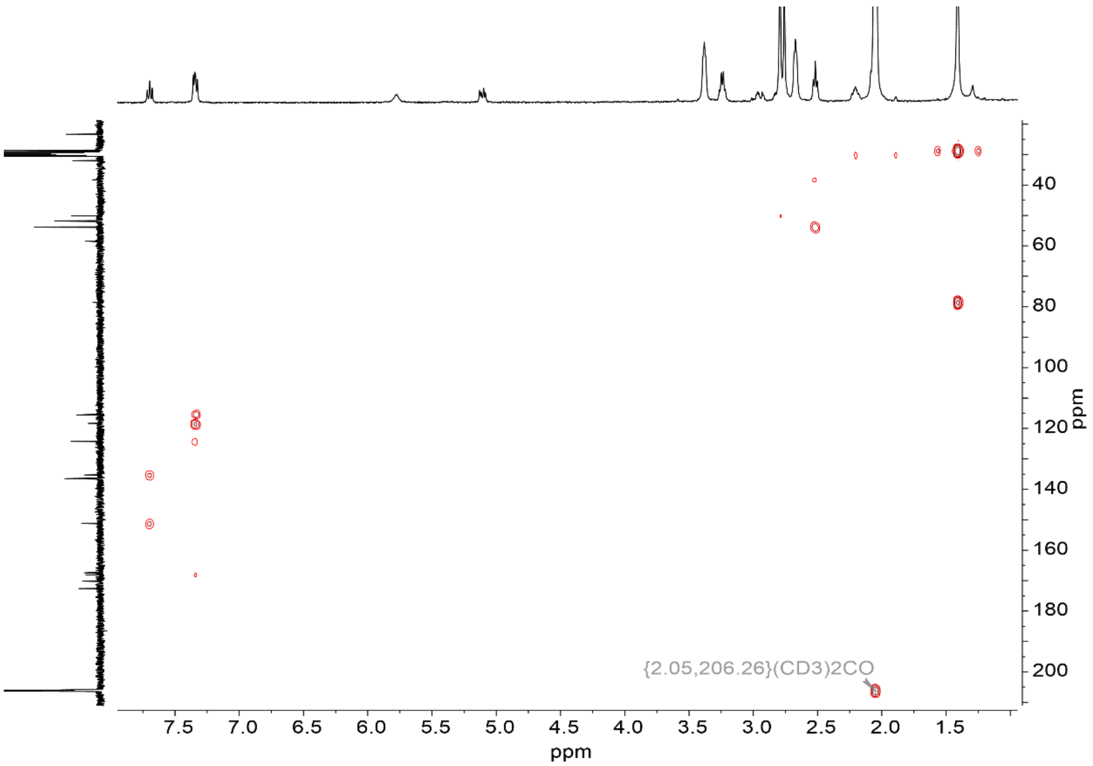

Figure S4. ^{1}H-^{13}C HMBC NMR spectrum of **3** in acetone-d_6.

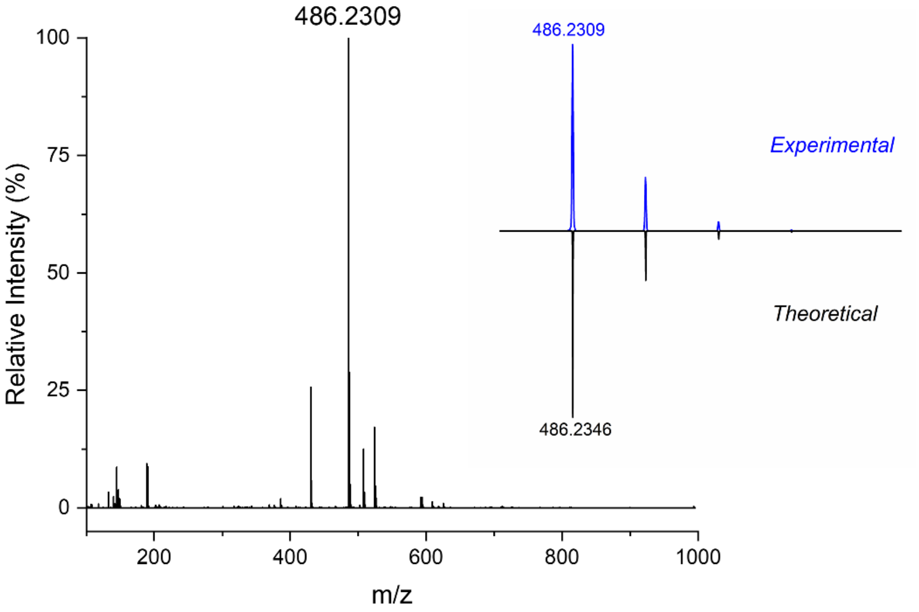

Figure S5. HR-ESI-MS spectrum of **3**: the inset shows the isotopic pattern of the species $[M+H]^+$ (m/z 486.2309, mass error = -7.609 ppm).

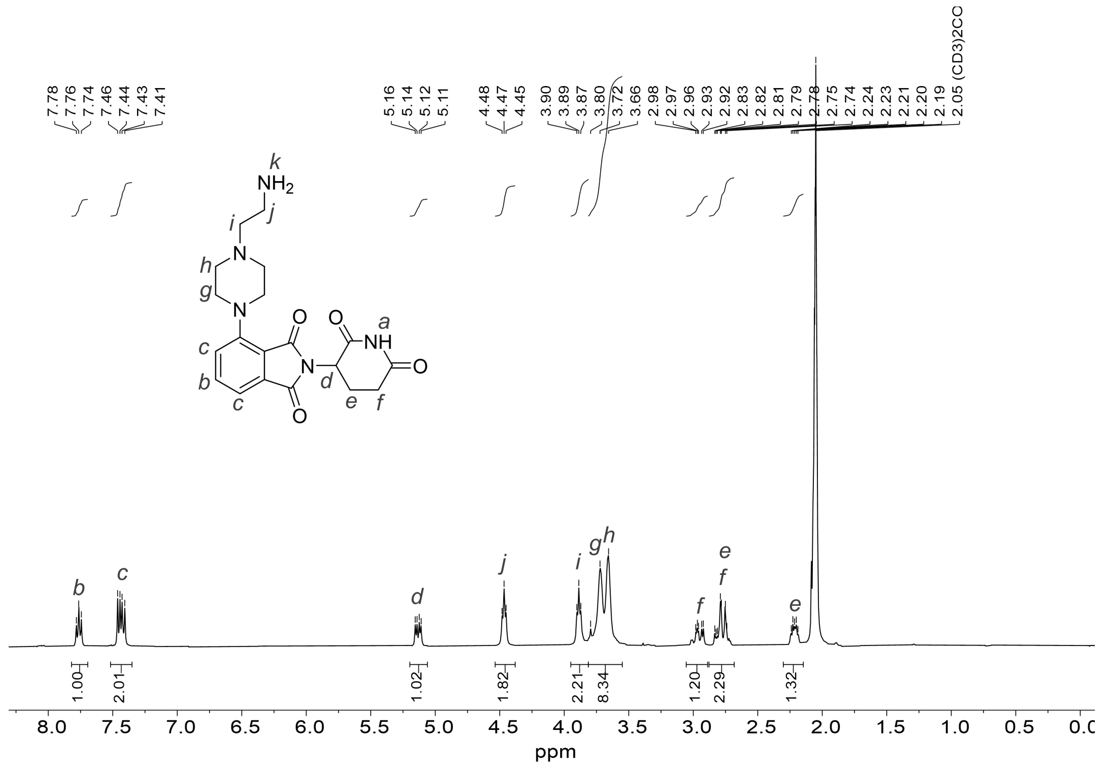

Figure S6. ^{1}H NMR spectrum (400 MHz) of **4** in acetone-d_6.

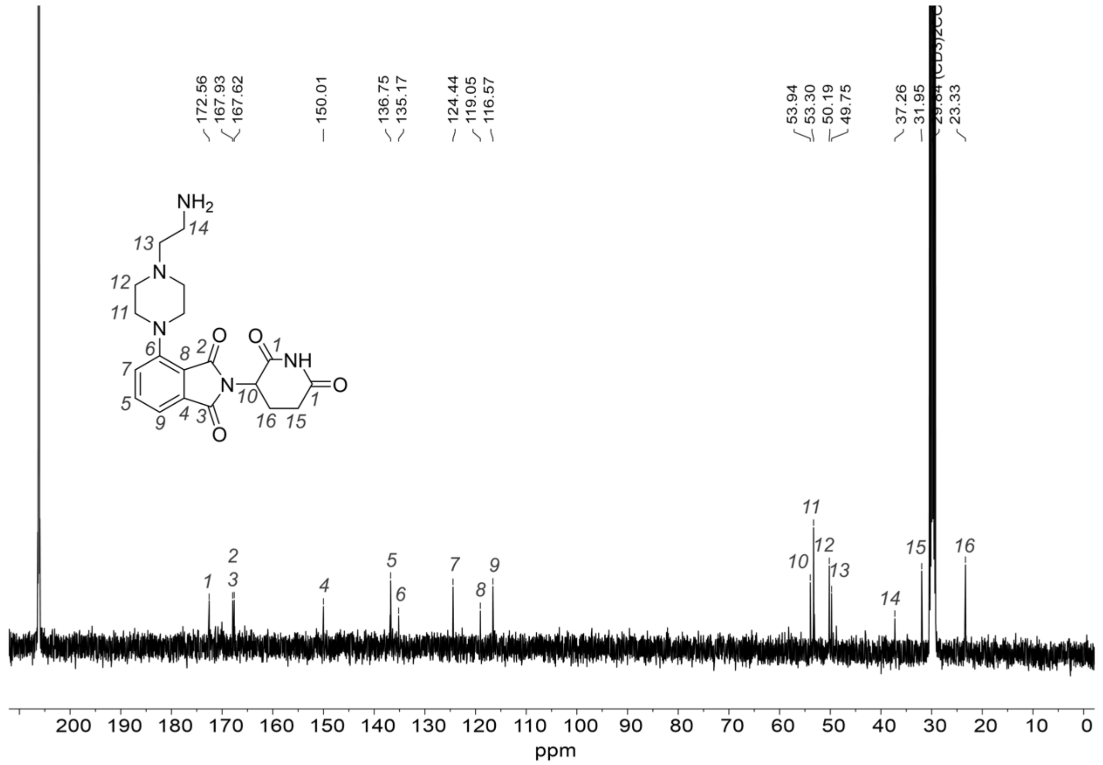

Figure S7. ^{13}C NMR spectrum (101 MHz) of **4** in acetone-d_6.

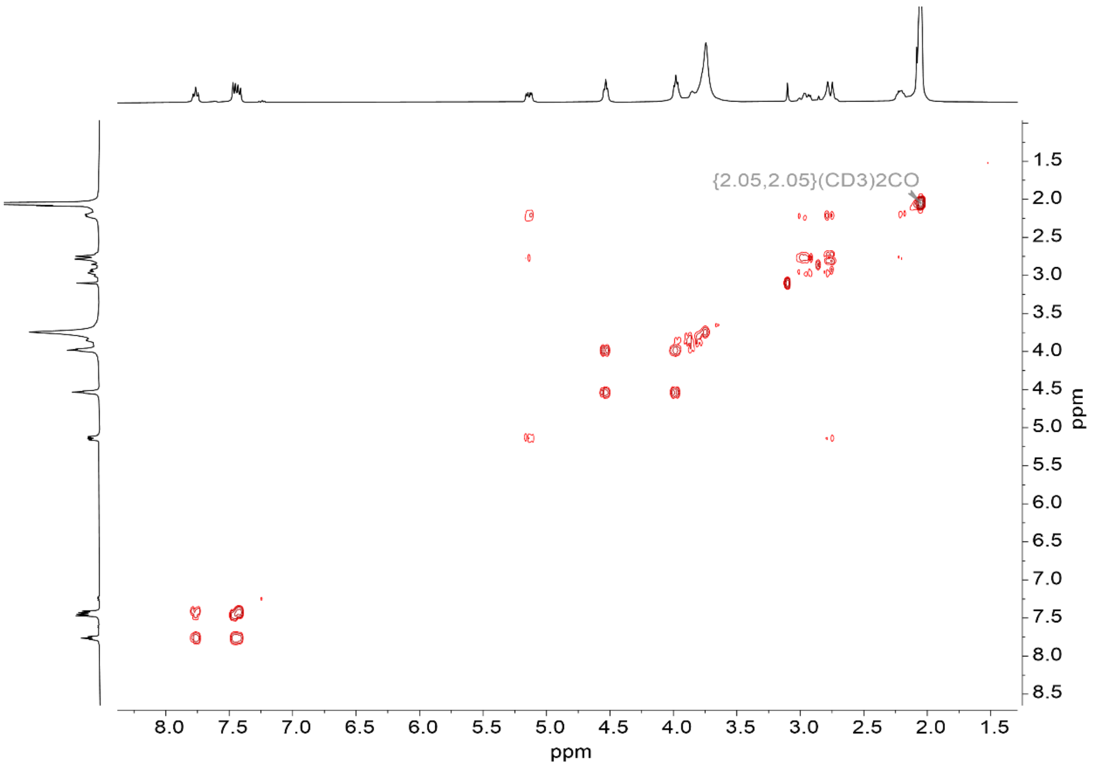

Figure S8. ^{1}H-^{1}H COSY NMR spectrum of **4** in acetone-*d*$_6$.

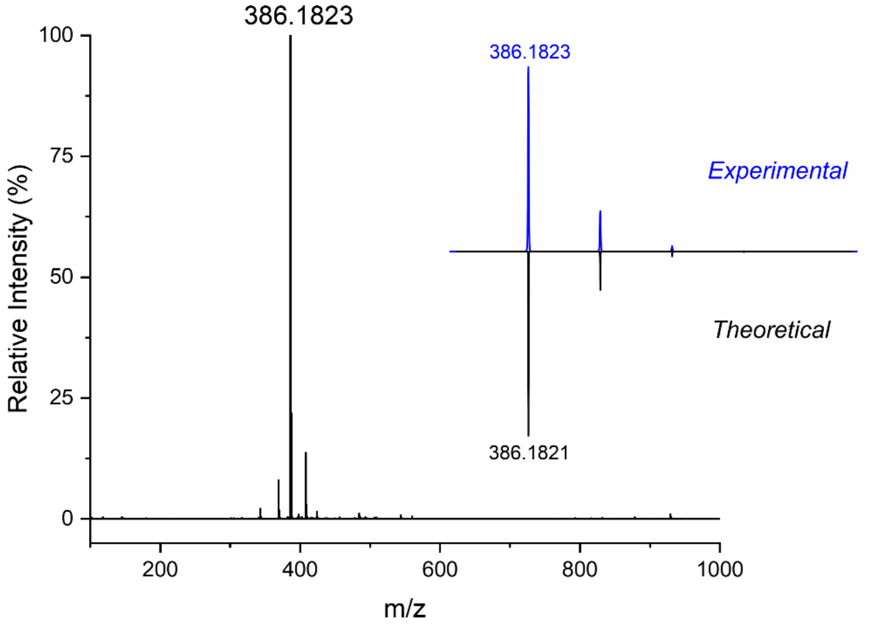

Figure S9. HR-ESI-MS spectrum of **4**: the inset shows the isotopic pattern of the species [M+H]$^+$ (m/z 386.1823, mass error = 0.5179 ppm).

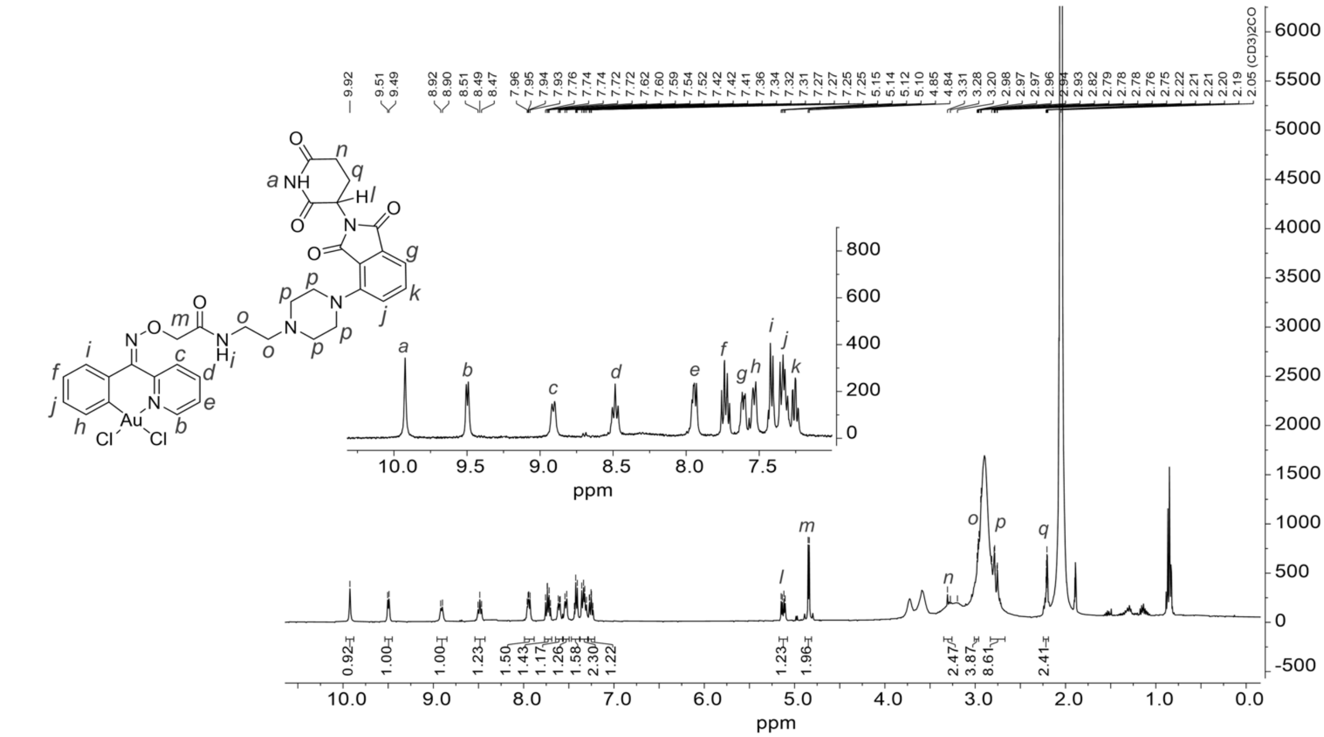

Figure S10. ^{1}H NMR spectrum (400 MHz) of **AuPROTAC** in acetone-d_6.

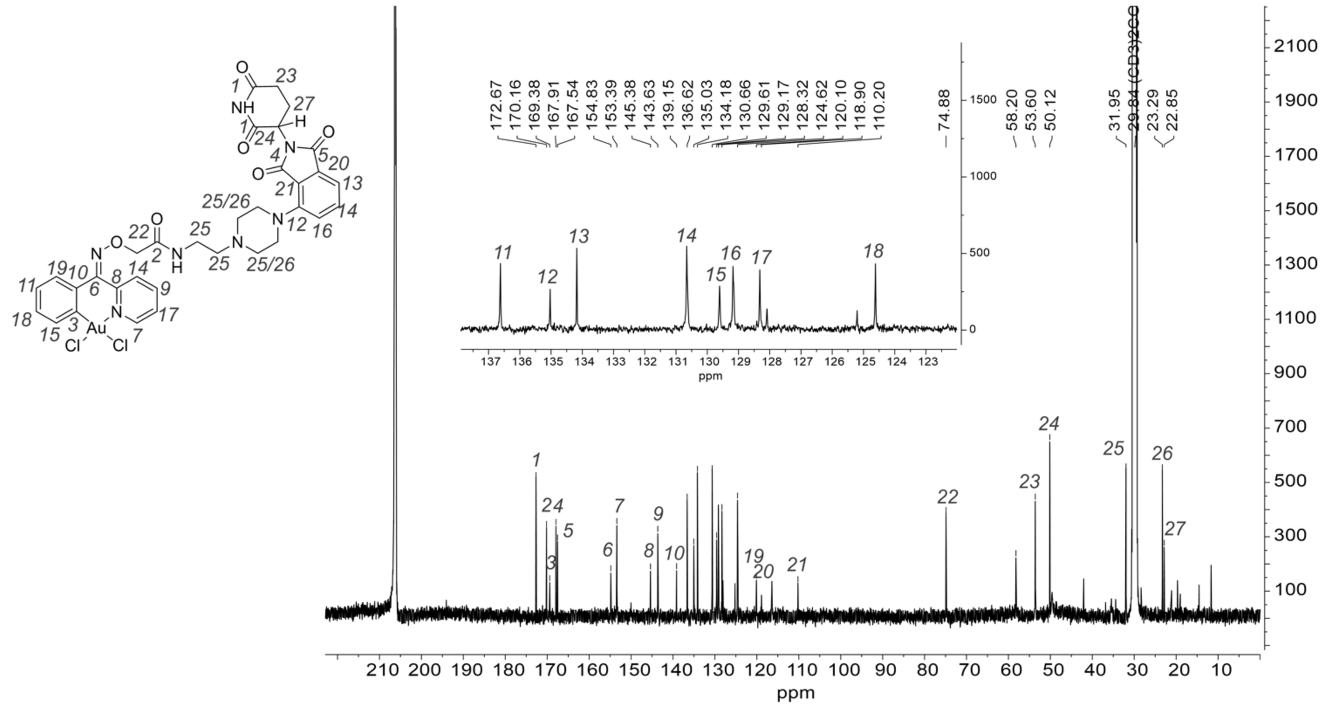

Figure S11. ^{13}C NMR spectrum (126 MHz) of **AuPROTAC** in acetone-d_6.

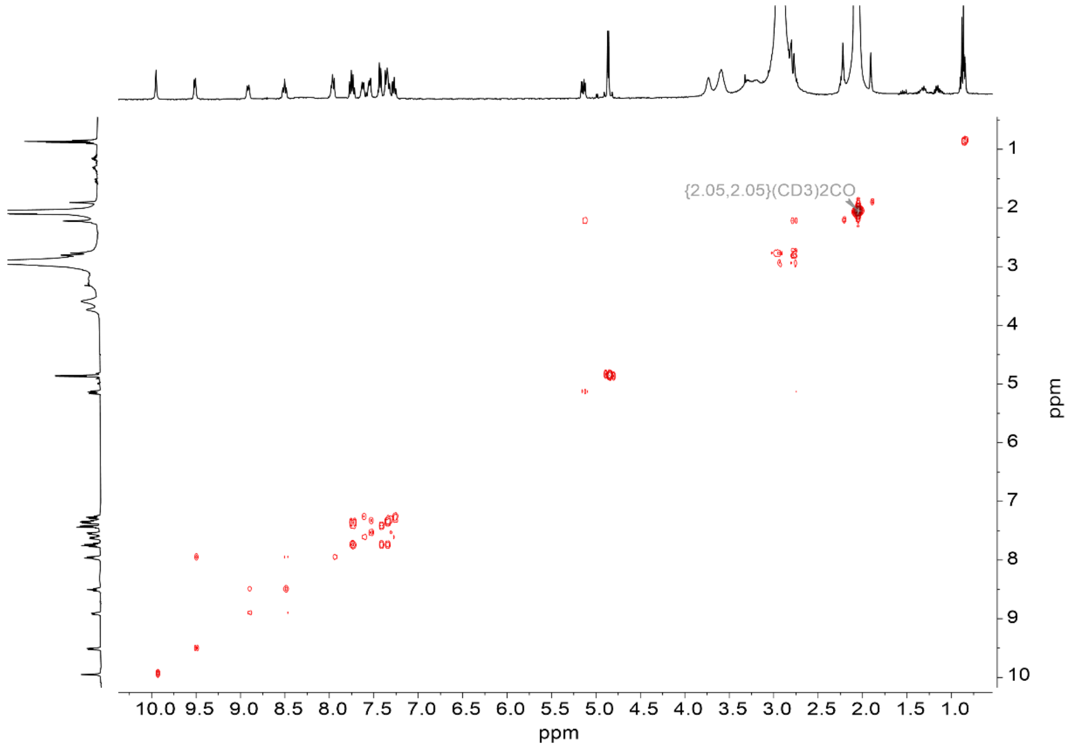

Figure S12. ^1H-^1H COSY NMR spectrum of **AuPROTAC** in acetone-d_6.

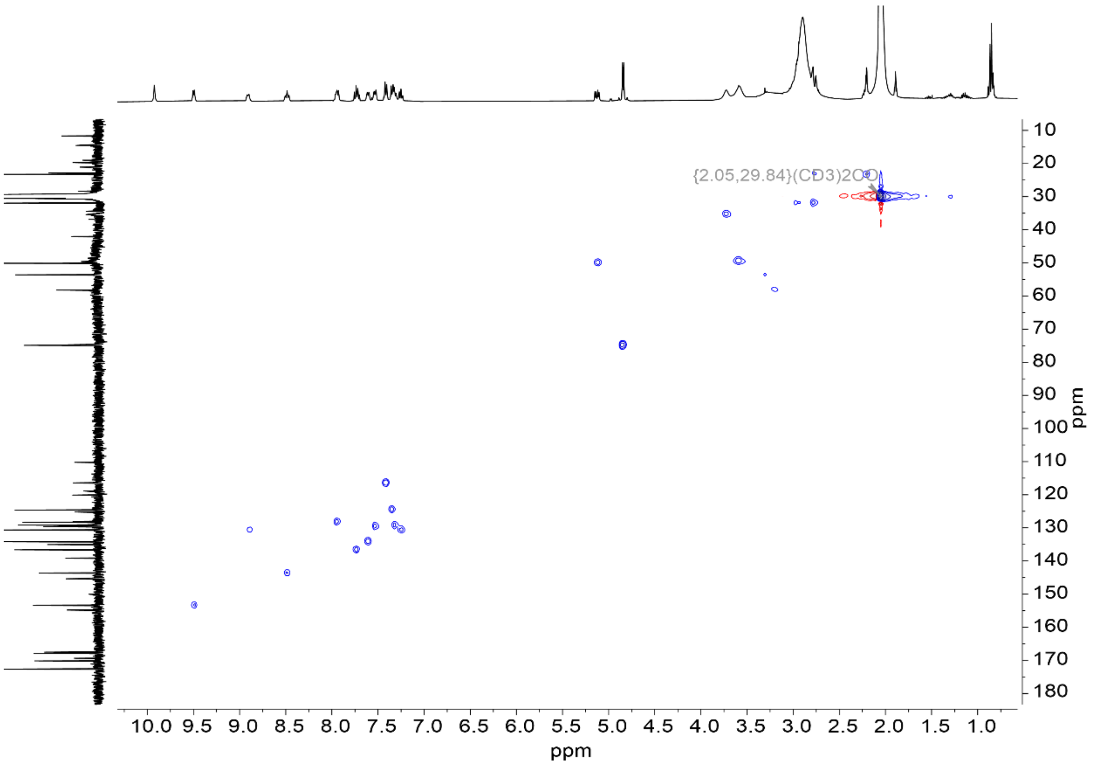

Figure S13. ^1H-^{13}C HSQC NMR spectrum of **AuPROTAC** in acetone-d_6.

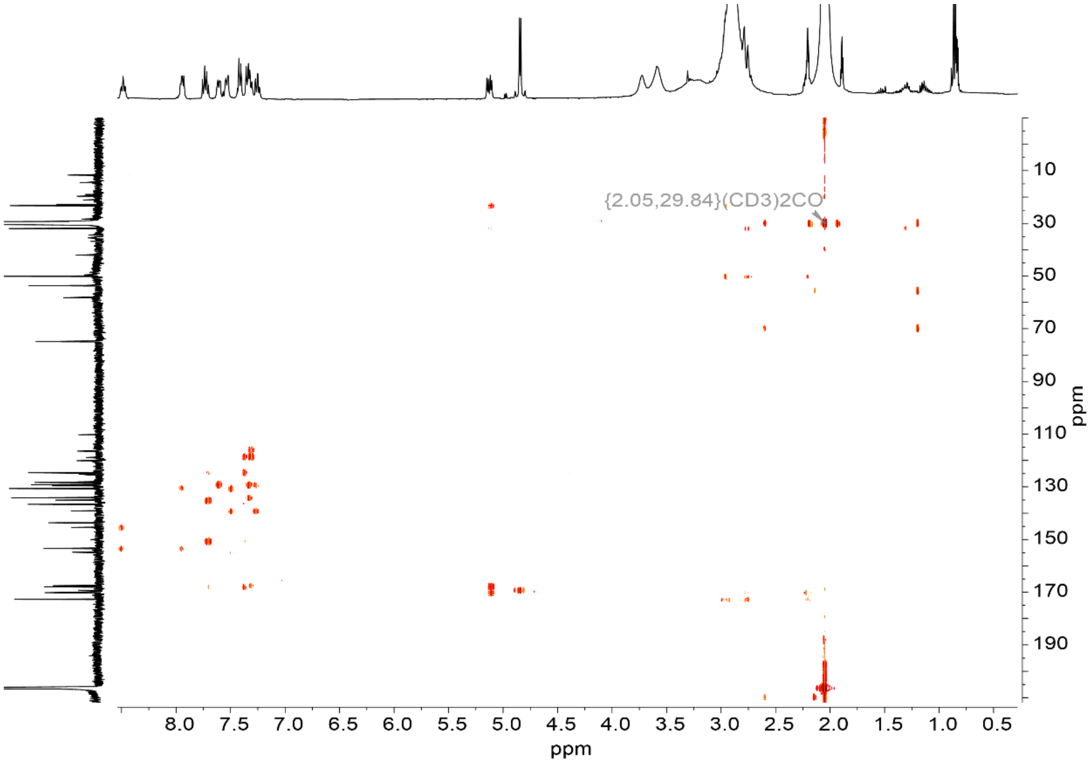

Figure S14. ^{1}H-^{13}C HMBC NMR spectrum of **AuPROTAC** in acetone-d_6.

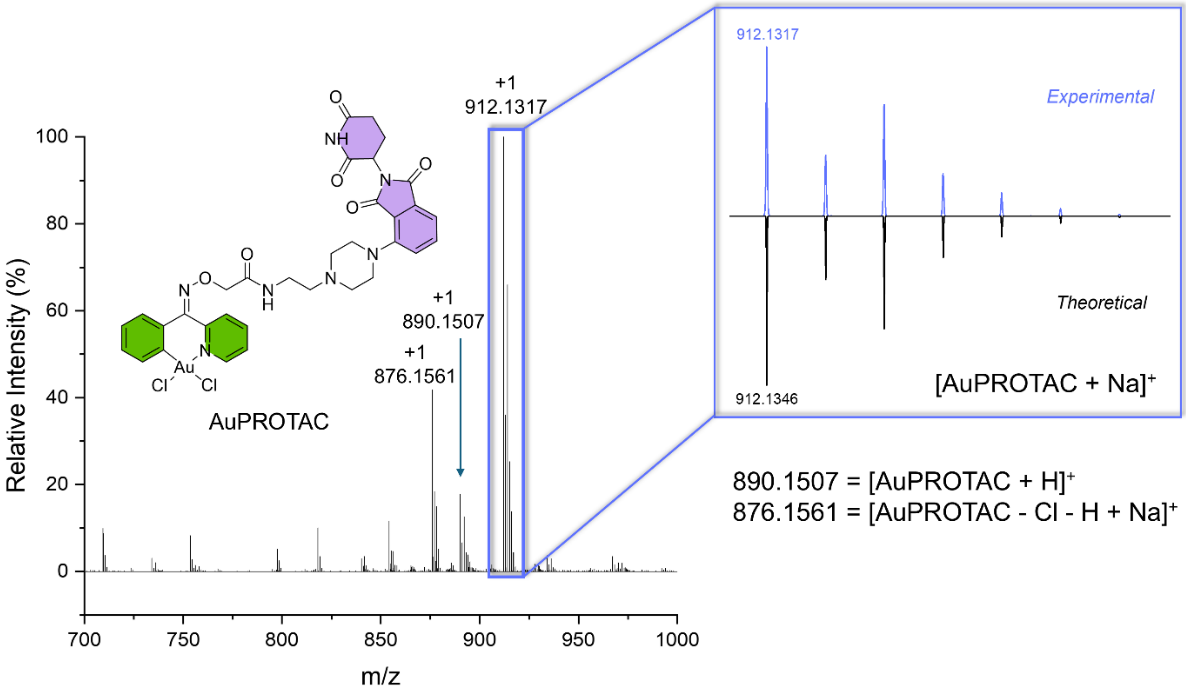

Figure S15. HR-DESI-MS of **AuPROTAC**: the inset shows the isotopic pattern of the species $[M+Na]^{+}$ (m/z 912.1317, mass error = -3.1794 ppm).

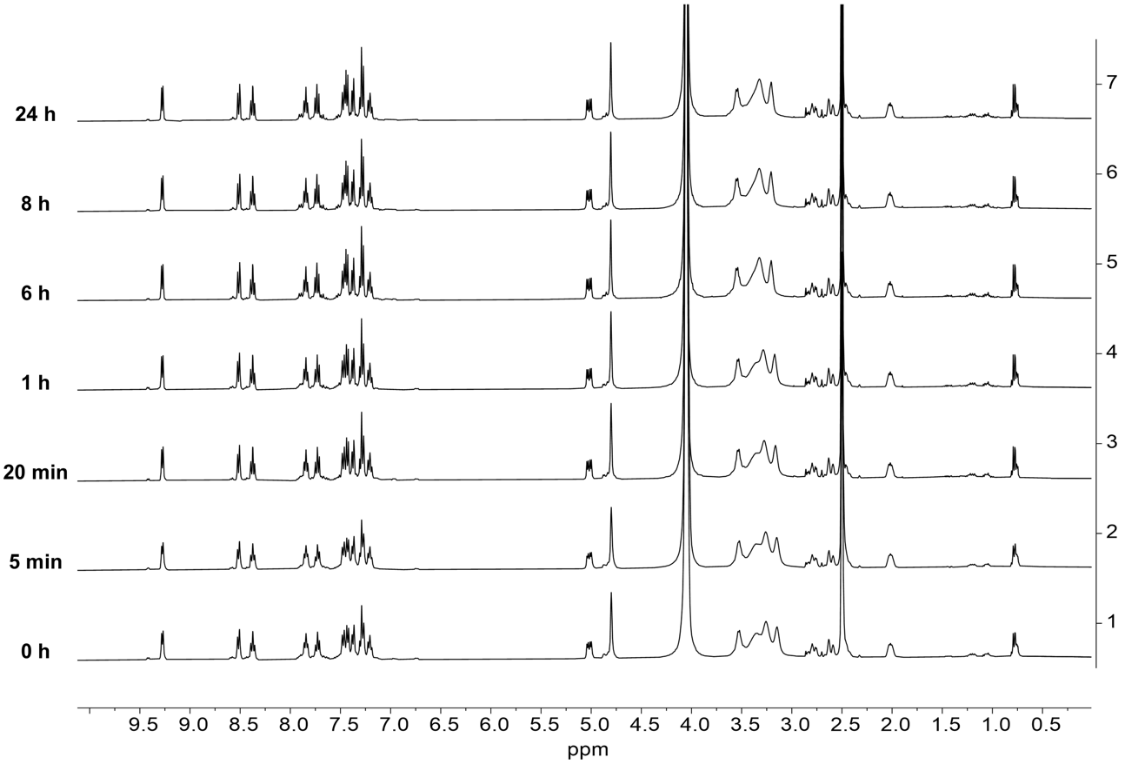

Figure S16. ^{1}H NMR stability kinetics for **AuPROTAC** in DMSO-d_6:D_2O (9:1) over 24 h.

A.

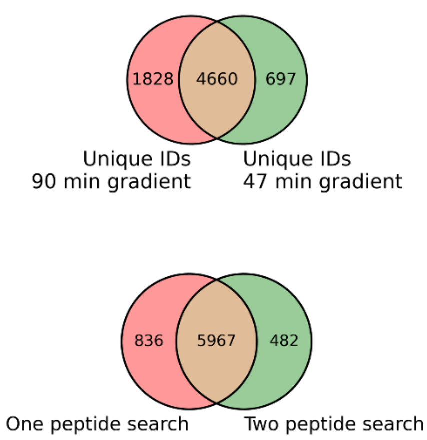

B.

47 min gradient		
Gene names	N° Peptides	N° Unique peptides
ABCC1	29	28
VHL	2	2
SMARCA2	0	0
SMARCA4	33	18
RBX1	2	2
CUL2	35	35
CUL4A	49	38
CUL4B	31	20
TCEB1	4	4
TCEB2	8	8
DDB1	50	50
TXNRD1	23	22
PBRM1	0	0
CRBN	8	8
ABCB10	19	18

90 min gradient		
Gene names	N° Peptides	N° Unique peptides
ABCC1	31	31
VHL	3	3
SMARCA2	34	17
SMARCA4	39	22
RBX1	3	3
CUL2	34	34
CUL4A	49	38
CUL4B	38	27
TCEB1	5	5
TCEB2	10	10
DDB1	52	52
TXNRD1	25	1
PBRM1	51	50
CRBN	12	12
ABCB10	22	21

47 min gradient	
N° Peptides	N° Unique peptides
13.7 ± 12.6	12.4 ± 11.9

90 min gradient	
N° Peptides	N° Unique peptides
16.1 ± 13.3	14.7 ± 12.7

C.

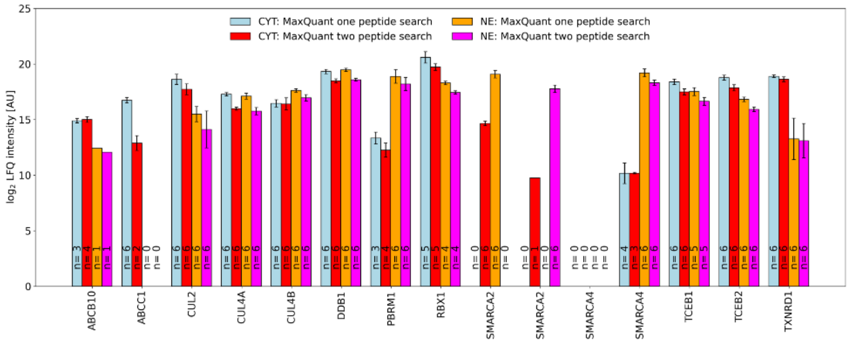

D.

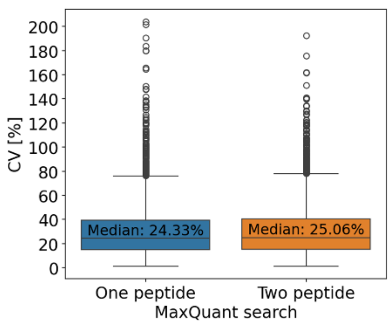

E.

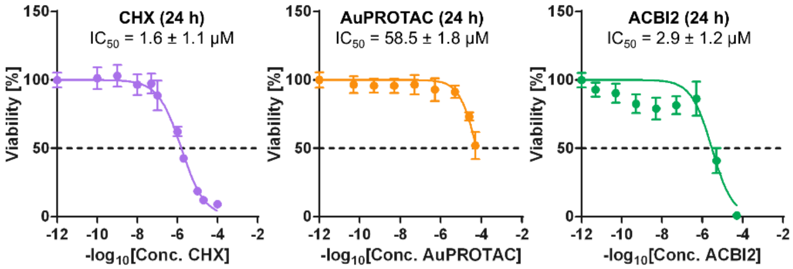

F.

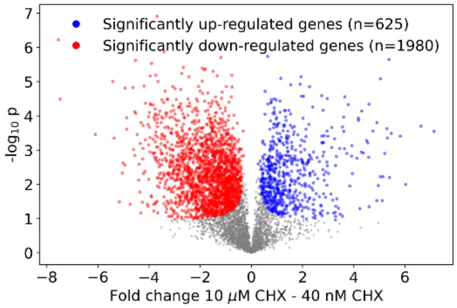

G.

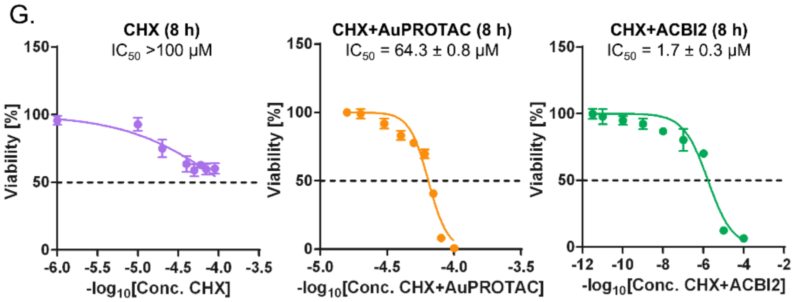

Figure S17. A. Venn diagrams of (*top*) the identified protein groups in HL-60 cells using a 47 min and a 90 min chromatography gradient during data acquisition and (*bottom*) the identified protein groups according to searches

requiring a minimum of 1 or 2 unique peptides. **B.** Comparison of the number of peptides and unique proteins for proteins of interest using the 47 min and 90 min gradients. **C.** LFQ-intensities of proteins of interest according to nucleocytoplasmic fractionation of HL-60 cells and according to searches requiring a minimum of 1 or 2 unique peptides for identification. **D.** The distribution of coefficients of variation (CVs) is shown as boxplots in the datasets of 1 or 2 unique MaxQuant searches. **E.** Cell viability assessments to determine the concentration to inhibit 50% growth (IC_{50}) in PMA-differentiated HL-60 cells. PMA differentiation was carried out for 72 h and the treatment for another 24 h. Dose-response curves were obtained from triplicates of hexuplicates (CHX) and duplicates of hexuplicates (**AuPROTAC**, ACBI2). **F.** Volcano plot of PMA-differentiated HL-60 cells treated with 10 µM or 40 nM of the translation inhibitor Cycloheximide (CHX). Highlighted proteins are statistically significant after multiple-testing correction (FDR 0.05, S0 = 0.1). **G**. Cell viability assessments to determine the concentration to inhibit 50% growth (IC_{50}) in PMA-differentiated HL-60 cells. PMA differentiation was carried out for 72 h, and the treatment for another 8 h. Dose-response curves were obtained from hexuplicates.

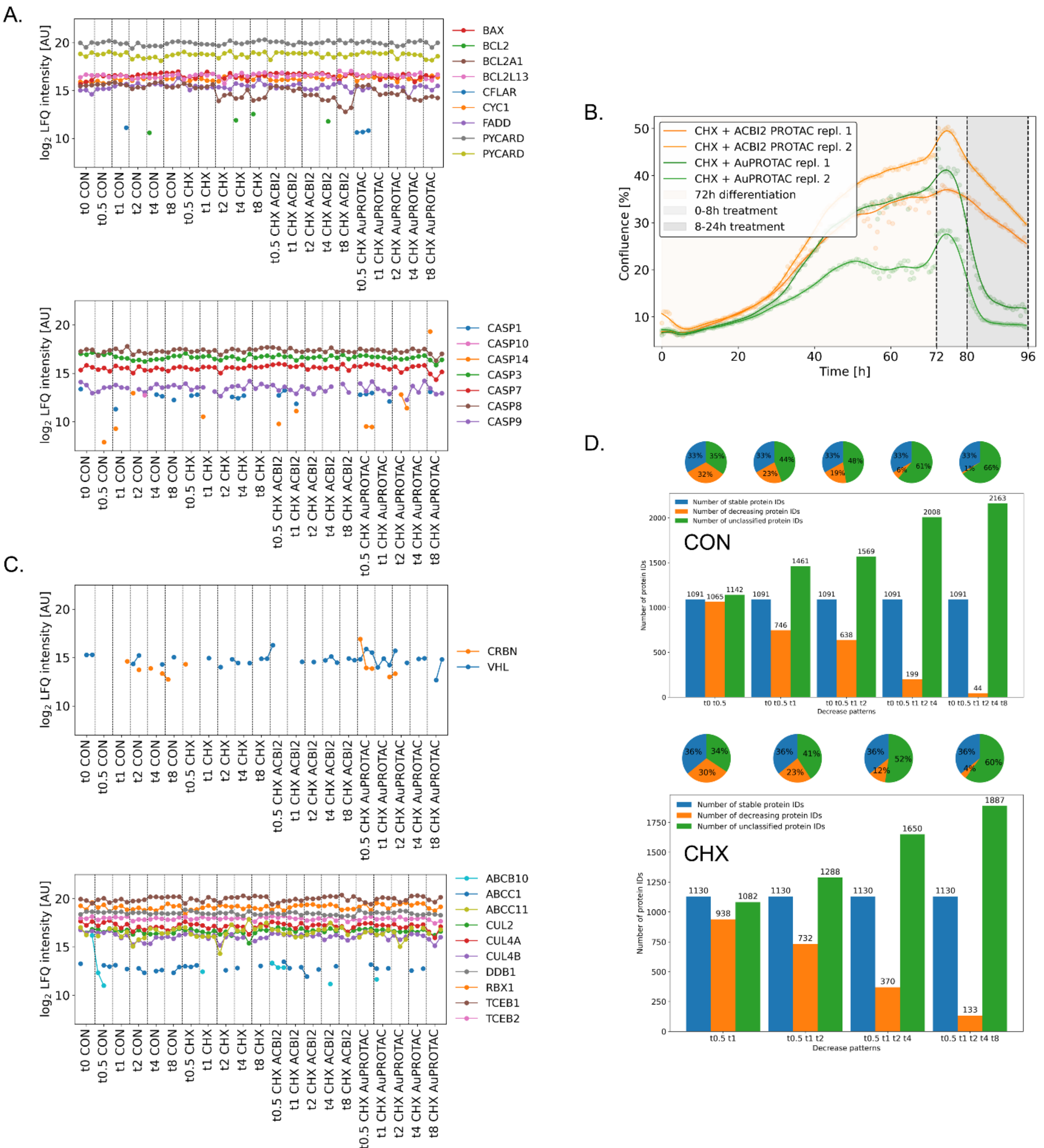

Figure S18. A. LFQ-intensities of apoptosis markers during the 8 h CHX+PROTAC chase. **B.** Time-dependent confluence plot of HL-60 differentiation with PMA and subsequent treatment with either the **AuPROTAC** or ACBI2. The 8 h interval used for the CHX+**AuPROTAC** chase assay is additionally highlighted. **C.** LFQ-intensities of proteins of interest for PROTAC activity over the course of the 8 h CHX+PROTAC chase. **D.** Dataset-level overview of stable (blue), decreasing (orange), and unclassified (green) according to stringency of selection in the controls (CON, *top*) and CHX-treated cells (*bottom*).

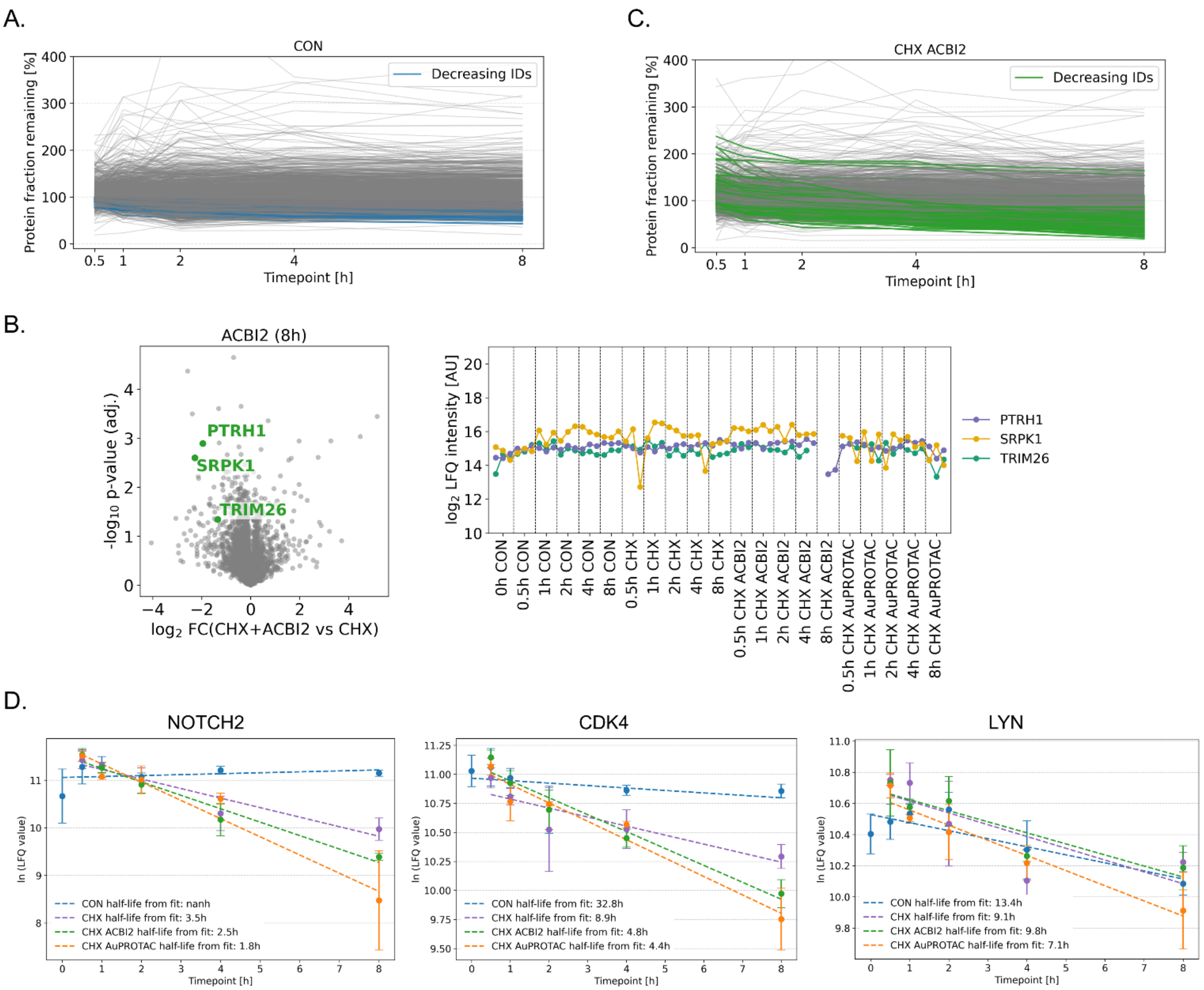

Figure S19. A. Profile plot of fully detected proteins (N = 3150) in CON cells as protein fraction remaining over 8 h incubation. The proteins highlighted in colour continuously decrease in intensity. **B**. Selection of the potential targets of ACBI2 by differential analysis of CHX+AuPROTAC vs CHX treatment after 8 h chase in Volcano and subsequent identification by degradation kinetics. Statistical significance of protein regulation shown in light coloured dots (FDR 0.05, S0 = 0.1). **C**. Profile plot of fully detected proteins (N = 3150) in CHX+ACBI2 cells as protein fraction remaining over 8 h incubation. The proteins highlighted in colour continuously decrease in intensity. **D**. Examples of protein half-life estimates from first-order decay kinetics to compare CHX and CHX+PROTAC activity.

Table S1. List of protein half-lives that were determined in both CHX and **AuPROTAC** conditions. The difference in protein half-live ($\Delta t_{1/2}$) is calculated as a measure of degradation efficiency.

Protein	CHX	CHX + AuPROTAC	$\Delta t_{1/2}$
FLNA	45,7	23,8	21,9
PIGR	21,0	10,5	10,5
LTV1	17,6	8,4	9,3
ARHGEF2	18,7	10,4	8,3
NCAM1	12,5	4,4	8,1
POLR2B	18,0	10,1	7,9
PPFIBP1	11,7	4,8	6,8
MT1X	13,9	8,0	6,0
NUMB	16,4	10,6	5,8
PPM1G	17,2	11,6	5,6
SPAST	14,2	9,0	5,3
ECE1	11,0	5,8	5,2
ZCCHC6	9,9	4,9	4,9
CDK4	8,9	4,4	4,6
ADAM10	10,0	6,0	4,0
NOSIP	9,5	5,9	3,7
SEC63	11,5	8,7	2,8
IVNS1ABP	7,4	4,7	2,7
NXF1	9,4	7,3	2,1
NOTCH2	3,5	1,8	1,6
PHAX	7,2	6,8	0,5
MTPAP	6,6	8,6	-2,1
CTTN	13,2	15,3	-2,1
DHX38	4,9	7,7	-2,8
HMOX1	5,6	8,4	-2,8
PRRC2C	4,2	7,2	-2,9
CEBPZ	8,7	12,1	-3,4

Table S2. List of protein half-lives that were determined in both CHX and ACBI2 conditions. The difference in protein half-live ($\Delta t_{1/2}$) is calculated as a measure of degradation efficiency.

Protein	CHX	CHX + ACBI2	$\Delta t_{1/2}$
SDHA	25,8	9,5	16,3
THUMPD3	20,7	8,0	12,7
NUP153	25,7	13,4	12,3
FLNA	45,7	34,9	10,8
XPO5	17,5	6,7	10,7
UBA6	34,3	24,7	9,6
EXOC1	22,6	13,0	9,6
UTRN	20,3	11,4	8,9
EIF3A	34,0	26,2	7,8
UNC13D	19,3	11,6	7,7
CTR9	12,9	6,5	6,4
RASAL2	20,0	13,9	6,1
PPP2CA	32,8	26,8	6,0
PPFIBP1	11,7	5,9	5,7
CASS4	11,0	5,3	5,7
ARHGEF2	18,7	13,7	5,0
HUWE1	12,1	7,8	4,3
ZCCHC6	9,9	5,7	4,2
CDK4	8,9	4,8	4,2
SMOX	21,1	17,4	3,7
CLCC1	9,3	5,7	3,5
BYSL	12,1	8,8	3,3
ADAM10	10,0	6,8	3,2
PHAX	7,2	4,5	2,7
GBF1	6,8	4,5	2,3
MYOF	6,2	4,0	2,3
CASP4	10,7	8,5	2,2
MKRN2	17,8	16,0	1,8
STAG2	5,3	3,6	1,7
IVNS1ABP	7,4	6,1	1,3
HMOX1	5,6	4,3	1,2
SEC63	11,5	10,3	1,2
HECTD1	7,7	6,6	1,1
MTPAP	6,6	5,5	1,1
NOTCH2	3,5	2,5	1,0
POLR2I	4,8	4,1	0,8
LONRF2	2,4	2,2	0,2
NUMB	16,4	16,3	0,1

Table S2. Continued.

Protein	CHX	CHX + ACBI2	$\Delta t_{1/2}$
SUN2	2,8	2,7	0,1
ITGB7	4,4	4,5	-0,1
SDF4	4,5	4,7	-0,2
MLF2	6,4	6,8	-0,4
CEP85	7,0	7,4	-0,4
CKAP5	8,1	8,8	-0,7
PRRC2C	4,2	5,0	-0,8
BIRC6	7,7	8,6	-0,9
TXNL4B	9,0	10,1	-1,0
STARD4	5,2	6,4	-1,2
CTTN	13,2	14,5	-1,4
RPS27	11,9	13,9	-2,0
PTPN22	11,9	13,9	-2,1
MTX1	21,3	24,6	-3,2
PHLDA1	4,9	8,2	-3,3
AGPAT9	7,6	12,1	-4,4
SNRPA1	15,1	31,0	-15,8
